# Dynamic Colonization of Microbes and Their Functions after Fecal Microbiota Transplantation for Inflammatory Bowel Disease

**DOI:** 10.1128/mBio.00975-21

**Published:** 2021-07-20

**Authors:** Nathaniel D. Chu, Jessica W. Crothers, Le T. T. Nguyen, Sean M. Kearney, Mark B. Smith, Zain Kassam, Cheryl Collins, Ramnik Xavier, Peter L. Moses, Eric J. Alm

**Affiliations:** a Center for Microbiome Informatics and Therapeutics, Broad Institute, Cambridge, Massachusetts, USA; b Department of Biological Engineering, Massachusetts Institute of Technology, Cambridge, Massachusetts, USA; c Graduate Program in Microbiology, Massachusetts Institute of Technology, Cambridge, Massachusetts, USA; d Larner College of Medicine, University of Vermont, Burlington, Vermont, USA; e Finch Therapeutics, Somerville, Massachusetts, USA; f Broad Institute, Cambridge, Massachusetts, USA; Brigham and Women’s Hospital/Harvard Medical School

**Keywords:** 16S RNA, bacteria, competition, fecal microbiota transplant, immunoglobulins, inflammatory bowel disease, metagenomics, microbiome

## Abstract

For fecal microbiota transplantation (FMT) to be successful in immune diseases like inflammatory bowel disease, it is assumed that therapeutic microbes and their beneficial functions and immune interactions must colonize a recipient patient and persist in sufficient quantity and for a sufficient period of time to produce a clinical benefit. Few studies, however, have comprehensively profiled the colonization and persistence of transferred microbes along with the transfer of their microbial functions and interactions with the host immune system. Using 16S, metagenomic, and immunoglobulin A (IgA) sequencing, we analyzed hundreds of longitudinal microbiome samples from a randomized controlled trial of 12 patients with ulcerative colitis who received fecal transplant or placebo for 12 weeks. We uncovered diverse competitive dynamics among donor and patient strains, showing that persistence of transferred microbes is far from static. Indeed, one patient experienced a dramatic loss of donor bacteria 10 weeks into the trial, coinciding with a bloom of pathogenic bacteria and worsening symptoms. We evaluated the transfer of microbial functions, including desired ones, such as butyrate production, and unintended ones, such as antibiotic resistance. By profiling bacteria coated with IgA, we identified bacteria associated with inflammation and found that microbial interactions with the host immune system can be transferred across people, which could play a role in gut microbiome therapeutics for immune-related diseases. Our findings shed light on the colonization dynamics of gut microbes and their functions in the context of FMT to treat a complex disease—information that may provide a foundation for developing more-targeted therapeutics.

## INTRODUCTION

Buoyed by early success in recurrent Clostridium difficile infections (1, 2), researchers are exploring whether fecal microbiota transplantation (FMT)—the transfer of entire fecal microbial communities from a healthy donor to a sick patient—can treat other microbiome-associated conditions. One of the most promising candidates is inflammatory bowel disease (IBD), a chronic condition characterized by periods of relapse (i.e., “flares”) and remission, which suggests that longitudinal dynamics are key to understanding and treating the disease (3). Compared with healthy individuals, patients suffering from IBD (ulcerative colitis or Crohn’s disease) have distinct gut microbial communities (4, 5). Thus, it has been hypothesized that manipulation of the gut microbiome and its interactions with the gut immune system might improve patient symptoms. Clinical trials have demonstrated that FMT is moderately effective in patients with ulcerative colitis (UC), but the factors driving patient response or nonresponse remain unknown (6).

It is broadly believed that the therapeutic element of FMT is microbes and their functions (7, 8). Many commensal bacteria are thought to promote gut and immune health, for example, by the production of butyrate, which plays metabolic (9), regulatory (10), and immune roles (11–14) in supporting the gut epithelium. But not all microbial functions are beneficial. Fecal transplant material is rigorously screened for pathogens, and large clinical studies have demonstrated fecal transplant’s broad safety (1), but the upsurge of antibiotic resistance has raised concerns that fecal transplants could transfer potentially deleterious microbial functions as well (15).

In addition to autonomous functions of the microbes themselves, the microbes’ interactions with the gut immune system may also play key roles in disease progression or treatment. A host’s immune system interacts with gut bacteria by responding to bacterial metabolites (12, 14), sensing direct contact between host epithelium and bacteria (16), and coating bacteria with immunoglobulin A (IgA)—the main antibody produced in the gut and other mucosal tissues (17). These interactions play a pivotal role in the formation and maintenance of the host’s immune system (18, 19). Since many microbiome-associated diseases—including IBD—are of immune origin, the gut microbiome’s immune function might be the most directly related to host health.

Despite excitement around applying fecal transplantation to IBD, no studies have comprehensively evaluated (i) which microbes transfer and persist across hosts, (ii) the microbial functions that accompany them, or (iii) whether immune functions of gut bacteria also transfer from donor to recipient. Previous reports of fecal transplants in IBD patients observed variable colonization by bacterial taxa from donors to recipients but did not categorize the functions and immune interactions that were also transferred (20–23). Furthermore, most of these studies used minimal sampling (e.g., single time points before and after a single fecal transplant) and so could not show how transferred bacteria and functions varied over time. Particularly in the case of chronic, inflammatory diseases such as IBD, understanding the longitudinal dynamics of transferred microbes and functions would advance our ability to determine why FMT works for some patients and not others and help pave the way for more-targeted therapies.

We comprehensively profiled the colonization dynamics of microbes and functions from a small randomized controlled clinical trial of 12 patients who had mild to moderate ulcerative colitis and were treated with FMT (24). By bringing together analysis of microbial taxa, strains, functions, and immune interaction in this focused clinical cohort, we sought to deeply understand colonization in a limited number of patients and reveal some of these dynamics in the context of a complex disease.

## RESULTS

### Study design.

**FIG 1:**
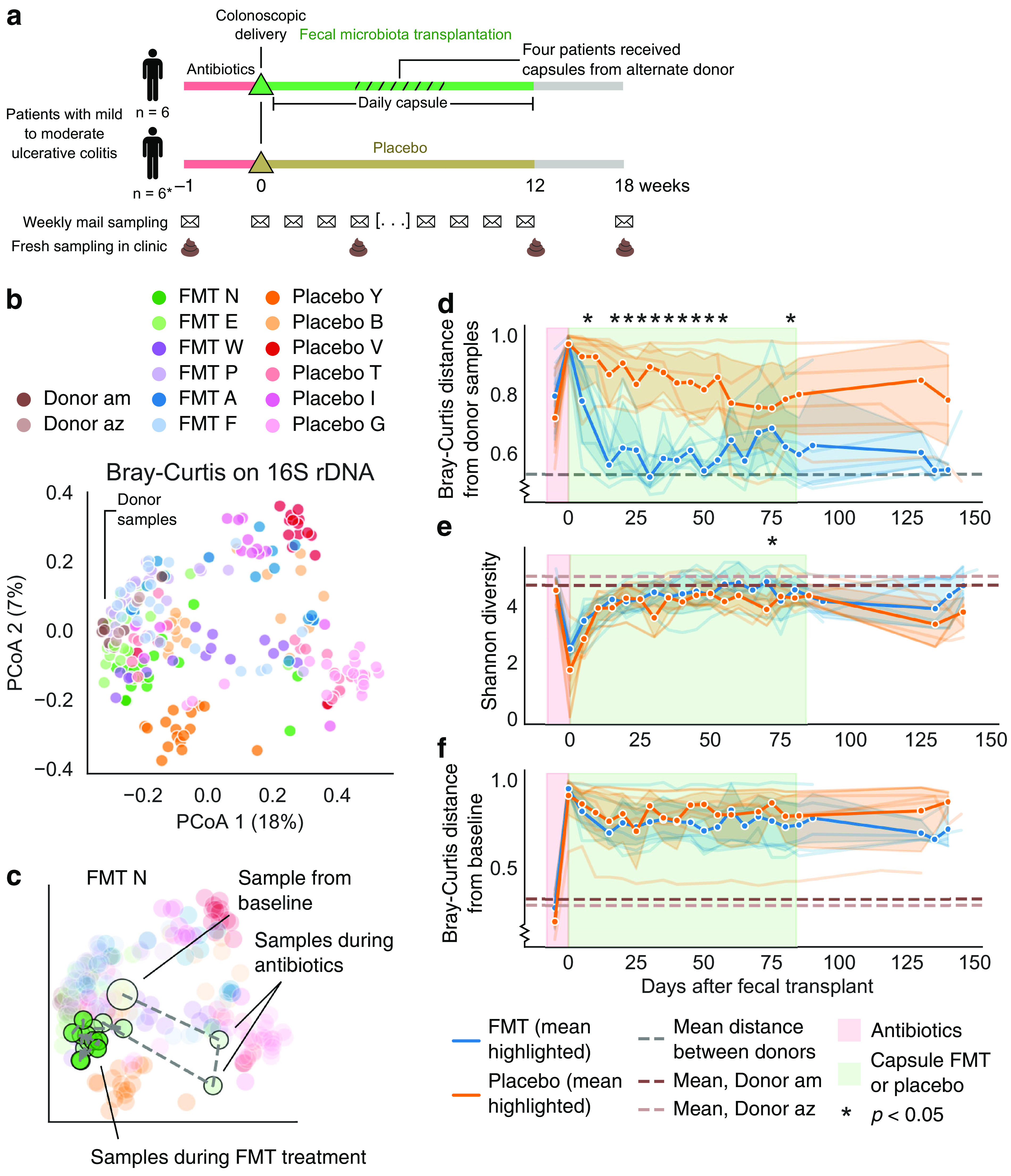
Fecal transplants resulted in global transfer and persistence of donor strains. (a) Design of the clinical trial and sampling. *, One placebo-treated patient had worsening symptoms and dropped out of the trial at 8 weeks. (b) Principal-coordinate analysis (PCoA) based on Bray-Curtis distance using 16S. Donor samples clustered on the left side. (c) Example patient trajectory over the course of the trial. Many patients demonstrated a strong shift in their gut microbiota in response to antibiotics and then an altered microbiota during and after FMT treatment (see Fig. S3). (d) Time series of each patient’s mean Bray-Curtis distance from the donor samples according to 16S data. FMT patients (blue) shifted toward donor communities (lower values on *y* axis). Bold lines and confidence intervals (95%) reflect the mean across patients. Lines for individual patients appear in the background. Asterisks indicate a significant difference between fecal transplant and placebo patients by a Mann-Whitney U test *P < *0.05. Regions of the graph colored as in Fig. 1. (e) Alpha diversity (Shannon index) of 16S profiles indicated little difference between fecal transplant and placebo recipients. (f) Similarly, the two treatment groups showed similar extents of community change when compared against their baseline samples by Bray-Curtis distance of 16S data. See also Fig. S2.

We obtained samples from a small clinical cohort recruited at the University of Vermont Medical Center (24). In brief, two fecal transplant donors were chosen for high stool butyrate content—measured by gas chromatography—because loss of butyrate-producing microbes has been associated with inflammation and IBD (25). After a course of broad-spectrum antibiotics (oral pills: 250 mg of ciprofloxacin twice daily and 500 mg of metronidazole three times daily for 7 days) and a standard bowel preparation, patients received colonoscopic delivery (120 ml at a concentration of 1 g of stool/2.5 ml in the cecum and terminal ileum), followed by 12 weeks of daily capsules, of either fecal transplant material (550 μl of FMT capsule or ∼0.5 g of stool from one of our two donors) or placebo (sham colonoscopic and capsule FMT designed to visually mimic FMT; summarized in Fig. 1a and Fig. S1 in the supplemental material; for details, see Crothers et al. [24]). The choice of antibiotic pretreatment with two transplant delivery methods was intended to maximize each patient’s exposure to donor material and to increase the likelihood that donor bacteria would successfully colonize their new host (24). A subset of fecal transplant recipients (*n *=* *4) received capsule fecal transplant material from an alternate donor for 4 weeks in the middle of the clinical trial, after which they returned to taking material from their original donor (Fig. 1a), providing an opportunity to test whether a previously established microbial community could be infiltrated by new bacteria from low-dosage capsules. Near-weekly preserved stool samples were collected by mail from these patients during the trial and an 18-week follow-up. At four time points (baseline before antibiotics and 4, 12, and 18 weeks after fecal transplant), fresh stool samples were also collected during clinical check-ins from a subset of patients (*n *=* *8, see the supplemental material).

10.1128/mBio.00975-21.1 FIG S1 Detailed clinical trial treatments for each patient. The unbalanced clinical trial design resulted from insufficient patient recruitment. (a) Five placebo patients received antibiotics followed by colonoscopic and capsule sham FMT. (b) Three FMT patients received antibiotics followed by colonoscopic and capsule FMT from the stool of Donor am. For 4 weeks in the middle of the capsule FMT period, the donor material was switched to stool from Donor az. (c) One FMT patient received antibiotics, followed by colonoscopic and capsule FMT from the stool of Donor az. For four weeks in the middle of the capsule FMT period, the donor material was switched to stool from Donor am. Because of an error in FMT processing, two FMT patients (d and e) received FMT material from only a single donor. Stool collection method (mailed samples stored in RNAlater or fresh samples stored in glycerol buffer) did not systematically alter microbiome diversity metrics. (f) We compared paired microbiome samples—instances when a fresh stool sample (stored in glycerol) and a mailed stool sample (stored in RNA later) were collected within a week of each other (excluding samples taken before and after antibiotics) or on the same day—to test whether stool collection method systematically altered alpha diversity (Shannon index). Lines connect paired samples and show no systematic bias. Download FIG S1, PDF file, 0.1 MB. Copyright © 2021 Chu et al. 2021 Chu et al. https://creativecommons.org/licenses/by/4.0/ This content is distributed under the terms of the Creative Commons Attribution 4.0 International license.

10.1128/mBio.00975-21.2 FIG S2 Related to Fig. 1: Fecal transplants resulted in global transfer and persistence of donor strains (a) PCoA of unweighted UniFrac distance of 16S. (b) PCoA of Bray-Curtis distances based on metagenomic species. (c) Mean Bray-Curtis distance from the donor samples based on metagenomic species. (d to f) Longitudinal changes in alpha diversity based on Shannon diversity of metagenomic species (d), ASV richness (e), and metagenomics species richness (f). (g) Bray-Curtis distance from baseline samples based on metagenomic species. We further examined diversity metrics of FMT responders and nonresponders. (h) Time series of each patient’s mean Bray-Curtis distance from the donor samples based on 16S data. (i) Alpha diversity (Shannon index) of 16S profiles. (j) Time series of each patient’s mean Bray-Curtis distance from their baseline sample based on 16S data. (k) Time series of each patient’s mean Bray-Curtis distance from the donor samples based on metagenomic data. (l) Alpha diversity (Shannon index) of metagenomic species. (m) Time series of each patient’s mean Bray-Curtis distance from their baseline sample based on metagenomic data. Download FIG S2, TIF file, 2.1 MB. Copyright © 2021 Chu et al. 2021 Chu et al. https://creativecommons.org/licenses/by/4.0/ This content is distributed under the terms of the Creative Commons Attribution 4.0 International license.

10.1128/mBio.00975-21.3 FIG S3 Individual patient trajectories through PCoA space. (a) PCoA of Bray-Curtis distances based on 16S, same as Fig. 1b. (b) Large example trajectory of patient FMT N, with three sample regions indicated: baseline samples (start), antibiotics samples (abx), and treatment samples (treat). (c) Trajectories for all other patients with the same samples marked. Download FIG S3, PDF file, 2.1 MB. Copyright © 2021 Chu et al. 2021 Chu et al. https://creativecommons.org/licenses/by/4.0/ This content is distributed under the terms of the Creative Commons Attribution 4.0 International license.

We sequenced DNA from these stool samples at the Broad Institute (Cambridge, MA) using 16S ribosomal DNA (rDNA) sequencing and shotgun metagenomic sequencing, producing data sets comprising an average of ∼250,000 16S rDNA sequences and ∼30.5 million metagenomic DNA sequences per sample (see Table S1). (Table S1 and all other supplementary tables are available at https://github.com/nathanieldchu/uc_fmt/tree/master/supplemental_materials.) To identify the abundance of different amplicon sequence variants (ASVs, akin to a bacterial species), we processed the 16S rDNA sequences using QIIME 2 (26) and DADA2 (27). To track the abundance of bacterial species, we processed the metagenomic sequences using Metaphlan2 (28). We did not observe any significant differences in overall microbial diversity metrics between stool samples collected in the clinic versus by mail (see Fig. S1f) and further found that both 16S and metagenomic sequencing resulted in highly correlated data sets across samples (Mantel test using Spearman’s correlation: *r *=* *0.837, *P < *0.001).

### Fecal microbiota transplantation shifts the gut microbiome of UC patients.

Global diversity metrics indicated robust transfer and persistent colonization of donor bacteria in patients who received a fecal transplant. From our PCoA analysis of beta diversity, we found that each patient’s samples tended to cluster (permutational multivariate analysis of variance [PERMANOVA] pseudo-F* *=* *9.55, *P < *0.001), samples from both donors clustered together (PERMANOVA pseudo-F* *=* *3.00, *P < *0.001), and patient-to-patient differences drove most of the variance in both 16S and metagenomic analyses (Fig. 1b; see also Fig. S2a and b). The gut microbiomes of fecal transplant recipients clearly shifted toward the donor’s microbiome community during the trial, as indicated by the decreasing Bray-Curtis distance from donor samples; those of placebo-treated patients did not (Fig. 1c; see also Fig. S2d). This difference persisted for the ∼150-day trial period, although not for every transplant patient. Crothers et al. (24) categorized three patients receiving fecal transplants as “responders” because they showed consistent clinical, endoscopic and histologic evidence of disease improvement (Fig. 1; see also Table S6) and categorized the other patients as “nonresponders” (24). Although the clinical cohort was not large enough for robust analysis comparing these patient populations, we observed no qualitative difference in donor similarity between the groups (see Fig. S2h to m). Indeed, individual patients had divergent trajectories during the clinical trial, with many patients experiencing a strong shift in their gut microbiota in response to antibiotics, and then patients appeared to variously shift back to their initial community structure, shift toward the donors, or even settle in a new community composition (see Fig. S3).

Unlike previous studies, ours did not find greater alpha diversity in fecal transplant versus placebo recipients. Shannon index and richness in 16S and metagenomic data were similar in both treatment groups over the study period (Fig. 1d; see also Fig. S2e and f), as was the change in bacterial community from baseline samples, according to the Bray-Curtis distance (Fig. 1e; see also Fig. S2g). The similarities between these groups appear to be driven by sizable shifts in the microbiomes of placebo patients (Fig. 1e), which resulted in microbiome community shifts comparable to those of FMT recipients. These results contrast with multiple studies’ reports of increased diversity and change in gut microbiome composition in fecal transplant recipients compared with placebo recipients in diseases such as C. difficile infection (29, 30). Although it is possible that our cohort was too small for us to observe a significant difference between placebo and FMT patients, the results exemplify how FMT and even antibiotics can have varied effects in different diseases.

### Transferred microbial taxa exhibited varied dynamics in fecal transplant recipients.

With such frequent sampling, we could next profile not only the microbial taxa that colonized transplant recipients but also their downstream dynamics, both of which likely underpin clinical response.

**FIG 2:**
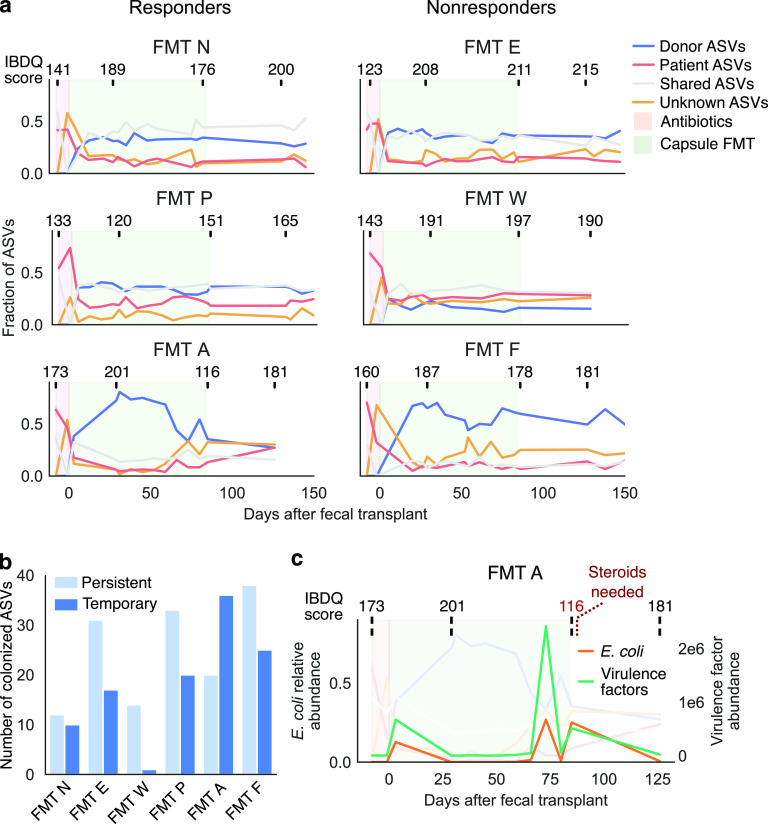
Longitudinal sampling of the microbiome revealed variable maintenance of donor bacteria. (a) Tracking bacterial sources identified bacteria transferred from donor to recipient, as well as an invasion of bacteria of unknown origin. Each time-series plot indicates the fraction of total ASVs (by 16S sequencing) identified as from the patient, from the donor, shared by the patient and donor, or from an unknown source. IBDQ scores reported by each patient are shown above each plot. Lower scores reflect worse disease, and higher scores reflect better health. Scores from patients in remission are usually above 170 to 190. Regions of the graph colored as in Fig. 1. (b) Fecal transplant recipients had various frequencies of persistent (retained at 18-week follow-up sampling after FMT) and temporary (not retained at 18-week follow-up) colonization of donor bacteria according to 16S data. (c) After an initial period of robust colonization, patient FMT A lost a majority of transferred donor bacteria; this loss coincided with a bloom of E. coli and accompanying virulence factors (based on metagenomic data) and a worsening of clinical symptoms. See also Fig. S4.

As in previous studies (31, 32), different patients in the trial varied in their colonization rates—that is, the number and frequency of donor bacteria that successfully transferred from donor to patient (Fig. 2a; see also Fig. S4 and Materials and Methods). We categorized bacteria in each patient’s samples by their putative sources, including those shared by the donor and patient before FMT (“Shared,” gray lines), those detected only in the patient’s baseline samples (“Patient,” orange lines), those detected only in the donor samples (“Donor,” blue lines), and those not detected (“Unknown,” yellow lines) (Fig. 2a; see also Fig. S4a). This final category potentially included newly colonized bacteria from the environment, as well as endogenous patient bacteria that were under our detection limit. At the resolution of metagenomic bacterial species and 16S ASVs, the proportion of bacteria transferred from the donor varied from 15 to 85% of a patient’s microbiome after fecal transplant (blue lines in Fig. 2a; see also Fig. S4a). Patients with larger numbers of donor-transferred bacteria were sometimes patients with fewer bacteria shared with the donor, but not always (e.g., FMT E and FMT P; Fig. S4b). Transferred bacteria spanned phylogenetic diversity, and almost all donor bacteria from one donor (referred to here as “Donor am”)—whose stool was transplanted into four recipients—were found to colonize at least one patient (see Table S4).

10.1128/mBio.00975-21.4 FIG S4 Related to Fig. 2: longitudinal maintenance of transferred bacteria. (a) Tracking bacterial sources identified bacterial species (based on metagenomic data) transferred from donor to recipient, as well as an invasion of bacteria of unknown origin. Each time-series plot indicates the fraction of total metagenomic species that were identified as from the patient or the donor, as shared between the two, or as from an unknown source. Regions of the graph are colored as in Fig. 1. (b) The counts of ASVs (based on 16S data) specific to the donor, specific to the patient at baseline, shared between the two, and categorized as transferred from the donor to the patient. This last category is a subset of the donor specific ASVs. (c) Persistent and temporary colonized bacteria based on metagenomic species. (d) Capsule delivery from an alternate donor introduced limited novel taxa. Tracking of ASVs as in Fig. 2 revealed only limited transfer of unique taxa from the alternate donor via daily capsules. The majority of donor-transferred ASVs were shared between the two donors, but ASVs specific to the induction donor tended to outnumber those from the alternate donor. Download FIG S4, PDF file, 0.3 MB. Copyright © 2021 Chu et al. 2021 Chu et al. https://creativecommons.org/licenses/by/4.0/ This content is distributed under the terms of the Creative Commons Attribution 4.0 International license.

Our analysis of longitudinal sampling further demonstrated that patients varied greatly in their ability to maintain colonized bacteria over time (Fig. 2). Most patients showed a period of initial colonization after fecal transplantation therapy began, followed by maintenance of transferred bacteria during the daily capsule delivery period and for months thereafter (Fig. 2a). In contrast, patient FMT A had robust colonization of donor bacteria early in treatment—in fact more so than any other patient—but later lost many of these bacteria (Fig. 2a). These colonized-then-lost bacteria included a number of ASVs of the genera *Bacteroides*, *Faecalibacterium*, *Ruminococcus*, and others (see Table S3). We categorized transferred ASVs in each patient as persistent or temporary colonizers on the basis of whether an ASV was present in follow-up samples (Fig. 1a). We found that different patients had different frequencies of these two types (Fig. 2b; see also Fig. S4c). Patient FMT A clearly had mostly temporary colonizers, but even patients with largely persistent colonization had taxa that colonized only temporarily.

Patient FMT A provides an intriguing clinical case: a sharp decrease in transferred donor bacteria coincided with a bloom of E. coli and associated virulence factors in this patient’s gut (Fig. 2c). In fact, the data suggested that the loss of donor bacteria may have preceded the bloom. Furthermore, these changes appeared to track clinical outcomes (Fig. 2c). The patient reported feeling better during the early stages of treatment (week 4)—as measured by the standardized, clinically validated Inflammatory Bowel Disease Questionnaire (IBDQ) that summarizes the patient’s symptoms over four domains of functioning and well-being (33)—but later reported worsening of symptoms (a flare at week 12), which required administration of a steroid (prednisone) (Fig. 2c). In addition, other clinical measures taken only at the beginning and the end of the trial (Mayo score, UCEIS, histology, lactoferrin, and calprotectin) also indicated a deterioration of disease over the course of the trial (see Table S6). Although we can only speculate whether the Escherichia coli bloom or the symptomatic change was the cause or the effect of losing donor bacteria, this case exemplifies the variability of bacterial persistence after FMT. We acknowledge that a single clinical case does not necessarily reflect a broader pattern, but we speculate that perhaps monitoring persistence of colonizing donor bacteria may help predict FMT treatment outcomes.

### Capsule delivery from an alternate donor introduced novel taxa into an established community.

By looking at the subset of patients who took capsules from an alternate donor partway through the study (Fig. 1a), we were able to ask whether changing donor material resulted in additional colonization by new microbes after a new gut microbiome had been established. Although the vast majority of newly colonized bacteria came from the original donor used for colonoscopic delivery and the first month of capsules, we were nonetheless able to identify evidence of novel bacteria colonizing from the alternate donor (see Fig. S4d). These results suggest that even after transplantation and establishment of a donor microbial community, UC patients remain receptive to further colonization and persistence of bacteria from additional donors.

### The balance of conspecific donor, patient, and environmental strains fluctuated between dominance and parity.

We then sought to profile the dynamics of individual strains within bacterial species to understand how conspecific strains (strains of the same bacterial species) from the donor, patient, and environment compete and coexist in treated patients. Previous reports have demonstrated that recipient and donor strains of the same bacterial species can coexist within fecal transplant recipients. Our analysis of frequent longitudinal samples allowed us to ask how the dynamics of this coexistence unfold over time. In particular, we sought to determine whether donor strains could dominate over patient strains—which, because they are endogenous, may have a competitive advantage—and whether the competitive balance of strains changes over time.

**FIG 3:**
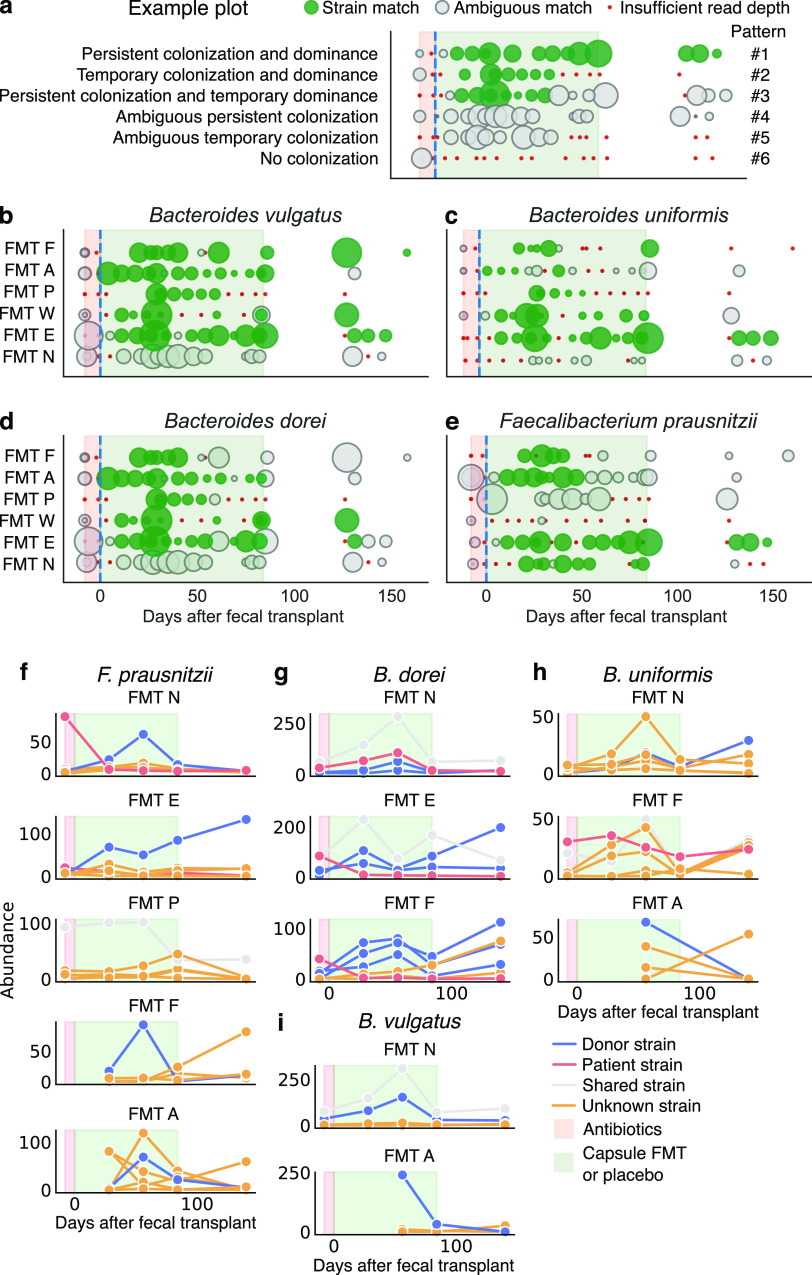
Tracking conspecific microbial strains revealed a range of competition dynamics. (a) Examples of plots for flexible genome analysis, which fell into six patterns determined by colonization and dominance. Green circles indicate a strain match between patient sample and donor sample, gray circles indicate ambiguous strain identity, and red circles indicate insufficient read depth for analysis. The size of the circle reflects the median read depth across the genome for that sample. (b to e) Flexible genome plots for Bacteroides vulgatus (b), B. uniformis (c), *B. dorei* (d), and F. prausnitzii (e). (f to i) In cases of ambiguous strain identities, we analyzed the individual contributions of strain haplotypes using StrainFinder for *F. prausnitzii* (f), B. uniformis (g), *B. dorei* (h), and B. vulgatus (i). The *y* axes represent the frequency of different strain haplotypes for a given species, normalized by the median read depth across all marker genes for that species (see Materials and Methods). See also Fig. S5.

We used two complementary strategies to evaluate the contributions of different strains to the resulting bacterial community in each patient: a flexible genome approach (high specificity, lower sensitivity) and a single-nucleotide-polymorphism (SNP) approach using StrainFinder (medium specificity, medium sensitivity) (31). We focused on bacterial species with high abundance and thus sufficient sequence read depth for robust analysis (Fig. 3a). Our flexible genome approach used read depth of flexible genomic regions to identify strains with identical gene content (34) and achieved high strain specificity by using full genome information. This approach can positively identify matches between samples with the same dominant strains (e.g., strain A versus strain A), but it cannot identify matches between samples that contain mixtures of strains (e.g., strain A versus strains A and B). Thus, a “mismatch” is considered ambiguous, since the two samples might contain entirely distinct strains or a mix of shared and distinct strains, resulting in lower sensitivity. Sample comparisons therefore had three different outcomes: strain match (green in Fig. 3), ambiguous (gray), and insufficient abundance or read coverage (red).

Using this flexible genome approach, we first confirmed that our methodology provided intuitively reasonable results by testing a number of control comparisons (e.g., donor samples did not match patient baseline samples, Fig. S5). We then compared samples from fecal transplant recipients to the donor’s samples and identified many matches between donor and patient samples after the start of FMT, indicating that the dominant strain in a given patient sample was the same as in the donor (Fig. 3a to d; see also Fig. S5c). We observed strain dynamics that fell into patterns determined by two factors: colonization (Did the donor strain colonize the recipient? No, temporarily, or persistently) and dominance (Did the donor strain become the dominant strain in the recipient? No, temporarily, or persistently). These factors resulted in six observed patterns, of which we provide examples in Fig. 3a.

1 *Persistent colonization and dominance*. The donor strain colonized after a fecal transplant, came to dominate the strain community, and persisted as the dominant strain throughout the trial. In Fig. 3a to e, this pattern appears as green circles that begin after fecal transplant and persist throughout the trial.
2 *Temporary colonization and dominance*. The same as pattern 1, but all strains were subsequently lost or undetected. In Fig. 3a to e, this pattern appears as green circles that dwindle to red dots.
3 *Persistent colonization and temporary dominance*. The same as pattern 1, but at later time points, another strain from the patient or from an unknown source became equally or more abundant than the donor strain. In Fig. 3a to e, this pattern appears as green circles that are later replaced by grey circles.
4 *Ambiguous persistent colonization*. Strains of this bacteria persistently colonized the patient, but it is unknown if those strains were from the donor, patient, or environment. In Fig. 3a to e, this pattern appears as grey circles that persist throughout the trial.
5 *Ambiguous temporary colonization*. Strains of this bacteria temporarily colonized the patient, but it is unknown if those strains were from the donor, patient, or environment. In Fig. 3a to e, this pattern appears as grey circles that dwindle to red dots.
6 *No colonization*. In Fig. 3a to e, this pattern appears as red dots.

10.1128/mBio.00975-21.5 FIG S5 Related to Fig. 3: strain-level transfer of donor bacteria. Using the flexible genome approach, we confirmed that samples from the same donor registered a strain match, while samples from different donors did not. Shown are the read depths of 1-kb windows of the reference genome for Faecalibacterium prausnitzii for two samples from the same donor (a) and two samples from different donors (b), with red circles indicating genome segments that were present in one sample, but absent in the other. (c) Flexible genome strain matches against donor samples for Bacteroides ovatus, *B. caccae*, B. fragilis, and *P. merdae* indicated further instances of transferred donor strains in fecal transplant recipients. To evaluate our flexible genome method, we confirmed that samples from placebo-treated patients did not match any of the donor samples (d), and samples from fecal transplant recipients who received an alternate donor did not match samples from that alternate donor except for isolated cases after the alternate donor period (e). (f to i) StrainFinder analyses of B. fragilis (f), B. thetaiotaomicron (g), Bifidobacterium longum, (h) and Eubacterium eligens (i). Download FIG S5, PDF file, 1.3 MB. Copyright © 2021 Chu et al. 2021 Chu et al. https://creativecommons.org/licenses/by/4.0/ This content is distributed under the terms of the Creative Commons Attribution 4.0 International license.

We observed examples of each of these patterns, indicating the range of competitive dynamics among strains that can unfold after fecal transplant. Many *Bacteroides* species were successful in persistently and dominantly colonizing (pattern 1), including B. vulgatus (patients E, W, and F), *B. dorei* (patients E and W), and B. uniformis (patient E) (Fig. 3b to d). The same bacteria in other patients persistently colonized but only temporarily dominated the patient community (pattern 2), including B. vulgatus in patient P, *B. dorei* in patients P and F, and B. uniformis in patients W and P. One patient (N) appeared to be more resistant to colonization by donor *Bacteroides* species, nearly always showing a mix of strains (pattern 4 or 6). We also observed temporary colonization and dominance for *B. ovatus* and *B. caccae* in fewer patients (see Fig. S5c). With regard to Faecalibacterium prausnitzii—a commensal bacterium thought to be related to gut health and negatively correlated with IBD (25)—some patients exhibited persistent colonization and dominance of a donor strain (pattern 1, patient E), others were only temporarily dominated by the donor strains (pattern 3, patients P and N), and still others adopted strains that came from both the donor and unknown sources (Fig. 3e).

In cases of ambiguous matches, our flexible genome analysis could not define the contributions of individual strains from different sources, leaving some competitive dynamics undefined. For example, a donor strain of *B. dorei* temporarily dominated the community of patient F, but later strain matching gave ambiguous results, which could mean that the donor strain disappeared from the community or that it coexisted with another strain (Fig. 3d). Consequently, to observe individual contributions of strains in mixed communities, our second approach reconstructed SNP haplotypes using StrainFinder, allowing us to distinguish contributions of strains from donor, recipient, and unknown sources over time. Although the sensitivity of StrainFinder allows us to quantify individual strains, its dependence on marker genes makes it less specific than our flexible genome approach (31). Because StrainFinder requires high sequencing depth to properly model strains, we combined longitudinal samples (*n *=* *1 to 5 samples) into five time points (see Methods and Methods and Fig. 3).

We found that donor and patient strains frequently coexisted, even after temporary dominance by the donor strain. We first confirmed that the results from StrainFinder aligned with our flexible genome analysis, as shown for *F. prausnitzii* in FMT N and FMT E (Fig. 3e). In the case of FMT A and FMT F, we observed that by the end of the clinical trial, strains of unknown origin largely displaced those from the donor (Fig. 3e). The dominant strains of some species (*F. prausnitzii* in FMT P and *B. dorei* and B. vulgatus in FMT N) were shared between the donor and patient (Fig. 3f to i). If we assume it is unlikely that two unrelated individuals carry identical strains, these results indicate that even high-resolution methods like StrainFinder depend on marker genes and cannot always resolve unique strains. For *B. dorei* in FMT F and B. uniformis and B. vulgatus in FMT A, donor strains temporarily dominated, with strains of unknown origin appearing in the patients’ follow-up samples (Fig. 3g to i). We observed a similar variety of competitive dynamics in other abundant species (see Fig. S5f to i).

Taken together, these results demonstrate not only that donor and recipient strains can coexist (32) but also that the balance of this coexistence changes over time. In many cases, donor strains were able to outcompete endogenous patient strains, but this dominance was dynamic, with donor and patient strains often competing with strains from unknown and possibly environmental sources later in the trial.

### Fecal transplants transferred beneficial microbial functions that varied across time.

**FIG 4:**
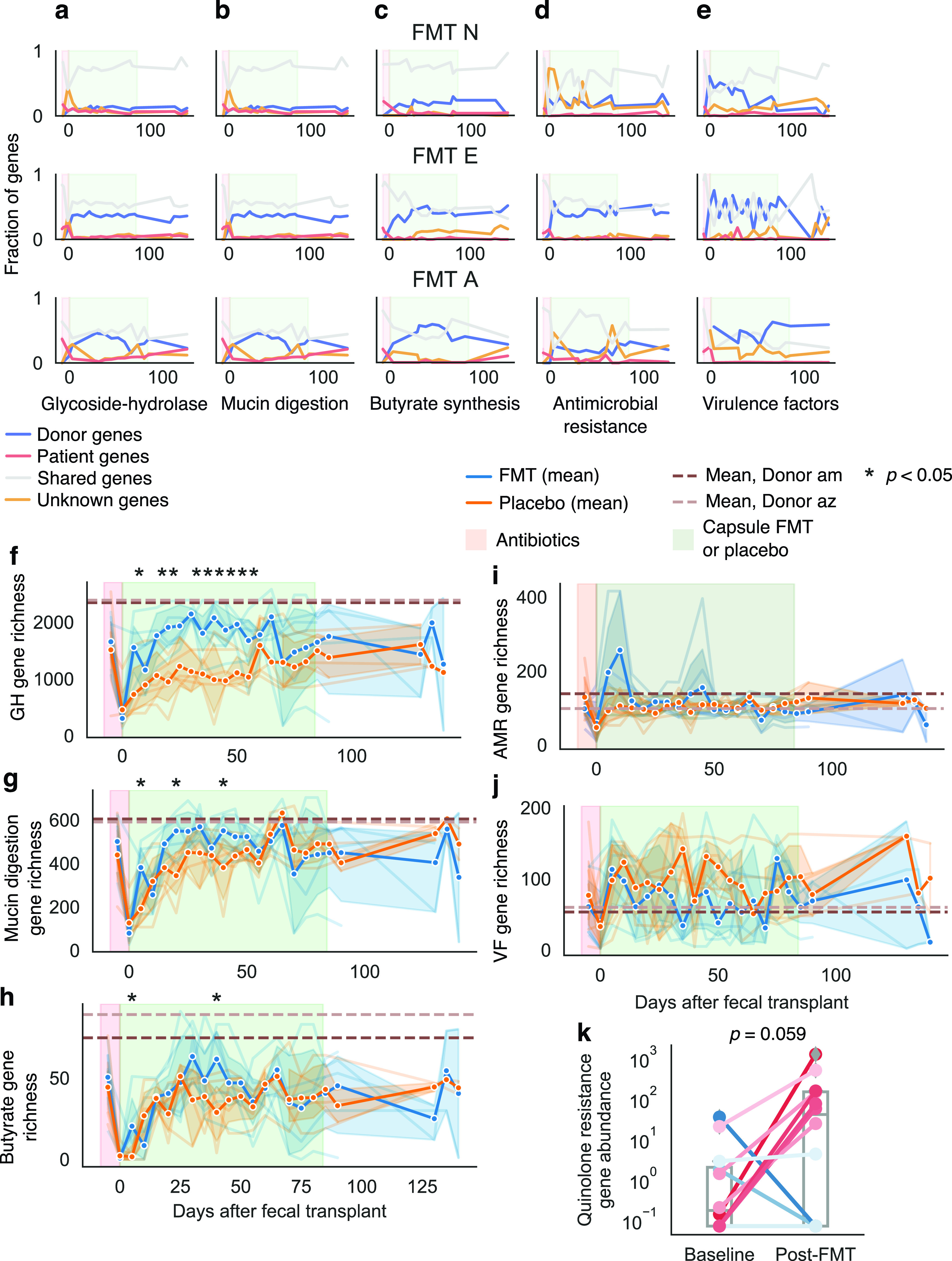
Bacterial functions also transferred across human hosts. We tracked the transfer of bacterial functions using shortBRED and curated databases (see Materials and Methods). (a to e) We observed colonization and persistence of functional genes in transplant recipients for glycoside hydrolase (GH) (a), mucin degradation (b), butyrate biosynthesis (c), antimicrobial resistance (d), and virulence factor (e) genes. Shown are plots for three of our transplant recipients (see Fig. S6). We also examined overall diversity of these genes compared with placebo-treated patients. (f to i) Fecal transplant recipients showed higher diversity of glycoside hydrolase genes (f) but not of mucin degradation genes (g), butyrate biosynthesis genes (h), or antimicrobial resistance genes (i). (j) Placebo patients had slightly higher diversity of virulence factors, but the difference was not significant. Asterisks indicate significant difference between fecal transplant and placebo recipients as determined by a Student *t* test *P < *0.05. (k) Many placebo and transplant recipients exhibited an increase in abundance of quinolone resistance genes in the 10 days after the administration of antibiotics. Lines are colored by the change in resistance. Line color reflects the change, with red lines indicating an increase, and blue lines indicating no change or a decrease. Also see Fig. S6.

Beyond the microbes themselves, the functions those microbes perform in the gut ecosystem may be important to restoring gut health. Therefore, we tracked colonization by functional genes implicated in maintaining health. We observed the transfer from donor to patient of genes involved in complex carbohydrate metabolism (glycoside hydrolase, Fig. 4a; see also Fig. S6a), mucin digestion (Fig. 4b; see also Fig. S6b), and butyrate production (Fig. 4c; see also Fig. S6c), and many of these genes persisted in their new hosts. These transferred genes included genes from health-associated commensals, such as *F. prausnitzii* and *Bacteroides* (see Table S5) (25, 35). Like the microbes themselves, these functional genes were transferred at different rates and had variable dynamics across patients (Fig. 4), and these dynamics largely mirrored patterns of microbial colonization (Fig. 2).

10.1128/mBio.00975-21.6 FIG S6 Related to Fig. 4: transfer of functional capacities. (a to e) Plots of the frequencies of microbial functions according to source for patients FMT W, FMT P, and FMT F for genes related to glycoside-hydrolases (a), mucin digestion (b), butyrate biosynthesis (c), antimicrobial resistance (d), and virulence factors (e). (f) Log_10_ cumulative abundance of all virulence factors in each patient across the trial period. (g) Cumulative abundance of virulence factors for all patients across the trial period. (h) Cumulative abundance of *Proteobacteria* in patient V during the trial period. Download FIG S6, PDF file, 0.7 MB. Copyright © 2021 Chu et al. 2021 Chu et al. https://creativecommons.org/licenses/by/4.0/ This content is distributed under the terms of the Creative Commons Attribution 4.0 International license.

Overall, fecal transplant recipients showed greater richness in glycoside hydrolase genes than did placebo-treated patients, suggesting increased capacity to digest dietary polysaccharides (Fig. 4f). On the other hand, fecal transplant recipients showed similar levels of butyrate biosynthesis (Fig. 4g) and mucin digestion genes (Fig. 4h). This, although donors were chosen for high butyrate production—reflected in high diversity of butyrate biosynthesis genes in donor samples (Fig. 4c, dashed lines)—fecal transplant did not result in wholesale transfer of these genes; transplant recipients had similar butyrate gene diversity compared with their baseline samples and with placebo-treated patients. Two fecal transplant recipients—but no placebo-treated patients—reached comparably high butyrate gene diversity, but these changes were temporary. Thus, fecal transplants can effectively transfer beneficial microbial functions across hosts but may not result in persistent colonization or an overall increase in the diversity of genes related to those functions.

### Fecal transplants also transferred antibiotic resistance and virulence factors.

Not all microbial functions transferring to fecal transplant recipients are beneficial. Although, clinically, fecal transplants can effectively clear patients of antibiotic-resistant infections, debate continues as to whether the therapy could introduce novel resistance genes, with negative clinical effects (15). We observed the transfer from donors to patients of numerous antibiotic resistance genes—including those for resistance to all major classes of antibiotics—and many of these genes were maintained for the full trial period (Fig. 4d; see also Fig. S6d, blue lines). Nevertheless, we found that the resulting diversity and abundance of antibiotic resistance genes in fecal transplant recipients did not exceed those in our healthy donors (Fig. 4i; see also Fig. S7a and b) and that transferred resistance genes were generally outnumbered by endogenous ones (Fig. 4d). Thus, although the transfer of resistance genes results unavoidably from the complexity of this therapy, no clinical or bioinformatic evidence indicates that fecal transplants increase the overall risk of antibiotic resistance. Indeed, many case reports suggest that FMT can help rid patients of antibiotic-resistant infections (36, 37).

In addition, we found that antibiotics triggered a short-lived increase in specific antibiotic resistance genes across all patients (Methods, Fig. 4k), regardless of treatment. Specifically, the abundance of quinolone resistance genes increased immediately after antibiotics, likely reflecting selection pressure from ciprofloxacin, a quinolone (Fig. 4k). These increases were not maintained over time (see Fig. S7c); neither did we observe temporary or lasting increases in other classes of antibiotic resistance (see Fig. S7c) or antibiotic resistance overall (see Fig. S7a and b). During the clinical trial, placebo-treated patients also had a greater burden of tetracycline and aminoglycoside resistance genes than did fecal transplant recipients during the clinical trial period, although these levels were not appreciably higher than in baseline samples (see Fig. S7d and e). We tracked the resistance profiles of taxa that included clinically significant pathogens and found that these resistance profiles generally followed the abundances of their bacterial host, with many resistance profiles peaking shortly after the administration of antibiotics (see Fig. S7f).

Similar to antibiotic resistance, we observed the transfer of virulence factors. Despite a lower incidence of virulence factor genes in donors than in patients (Fig. 4j), we observed colonization and persistence of such genes, which made up a significant portion of the virulence factor pool (Fig. 4e; see also Fig. S6e, blue lines). Many patients exhibited an increase in the abundance of virulence factors during or shortly after antibiotics (see Fig. S6f). Two patients had an inordinate burden of virulence factors, including the patient FMT A, who had a bloom of E. coli, and patient placebo V (see Fig. S6g). Patient placebo V’s health declined during the trial (see Table S6), and this patient had one of the most dramatic microbial turnovers in response to antibiotics (see Fig. S8)—resulting in a microbiome dominated by newly acquired *Proteobacteria* and associated virulence factors from unknown sources (see Fig. S6h). In sum, although we observed the transfer and persistence of donor-derived virulence factors and antibiotic-resistance genes, these transfers were modest and often outweighed by the endogenous microbial community’s own virulence and resistance.

10.1128/mBio.00975-21.7 FIG S7 Related to Fig. 4: longitudinal antibiotic resistance. (a and b) Cumulative abundance (a) and overall richness (b) of antimicrobial resistance genes in patients did not differ between placebo and fecal transplant patients. (c) Abundance of antimicrobial resistance of various classes across the time series. (d and e) We did observe possible increased abundance of tetracycline (d) and aminoglycoside (e) antibiotic resistance genes in placebo-treated patients compared with fecal transplant recipients. Download FIG S7, TIF file, 1.8 MB. Copyright © 2021 Chu et al. 2021 Chu et al. https://creativecommons.org/licenses/by/4.0/ This content is distributed under the terms of the Creative Commons Attribution 4.0 International license.

10.1128/mBio.00975-21.8 FIG S8 Microbial turnover in placebo patients. (a and b) Source plots as in Fig. 2a for placebo patients based on ASVs (a) and metagenomic species (b). Download FIG S8, PDF file, 0.04 MB. Copyright © 2021 Chu et al. 2021 Chu et al. https://creativecommons.org/licenses/by/4.0/ This content is distributed under the terms of the Creative Commons Attribution 4.0 International license.

### IgA coating of gut microbes identified shared immune interactions with commensal and IBD-associated bacteria.

In the context of IBD, interaction with the host immune system is perhaps the most important microbial function. Although it is reasonable to expect that transferring microbes would also transfer their endogenous metabolic capacities, it is much less certain whether transferred bacteria will elicit similar immune interactions—particularly adaptive immune responses—in a new host with a different immune system.

To begin understanding host response to transferred gut bacteria, we used immunoglobulin A sequencing (IgA-seq) to profile bacteria coated with IgA antibodies (17). Secretory IgA is the primary antibody of mucosal surfaces, including the gastrointestinal, respiratory, and urinary tracts (38). Thought to act primarily by blocking proteins on the surface of invading pathogens, IgA has more recently been suggested to play a role in facilitating mucosal colonization by commensal bacteria (39, 40). Although bacteria interact with the host immune system in many ways (e.g., via excreted metabolites [12] or direct contact with the epithelium [16]), we used IgA-seq as a proxy for bacterial immune function and interactions because IgA coating is one of the few markers of immune interactions that can be measured *in vivo* and at high throughput for bacteria in stool samples. To identify IgA-coated and -uncoated bacteria, we used fluorescence-activated cell sorting (FACS) to separate these fractions of gut microbiome samples, and we 16S-sequenced the fractions (∼50,000 cells per fraction) to a median depth of 165,000 reads. We calculated IgA coating scores for each bacterium as the log-fold change in abundance of that bacterium in the IgA-coated and IgA-uncoated fractions, such that a positive value indicated high IgA coating and a negative value indicated low IgA coating. To quantify similarity in IgA coating between samples, we calculated the Pearson correlation of IgA coating scores across all bacterial ASVs.

**FIG 5:**
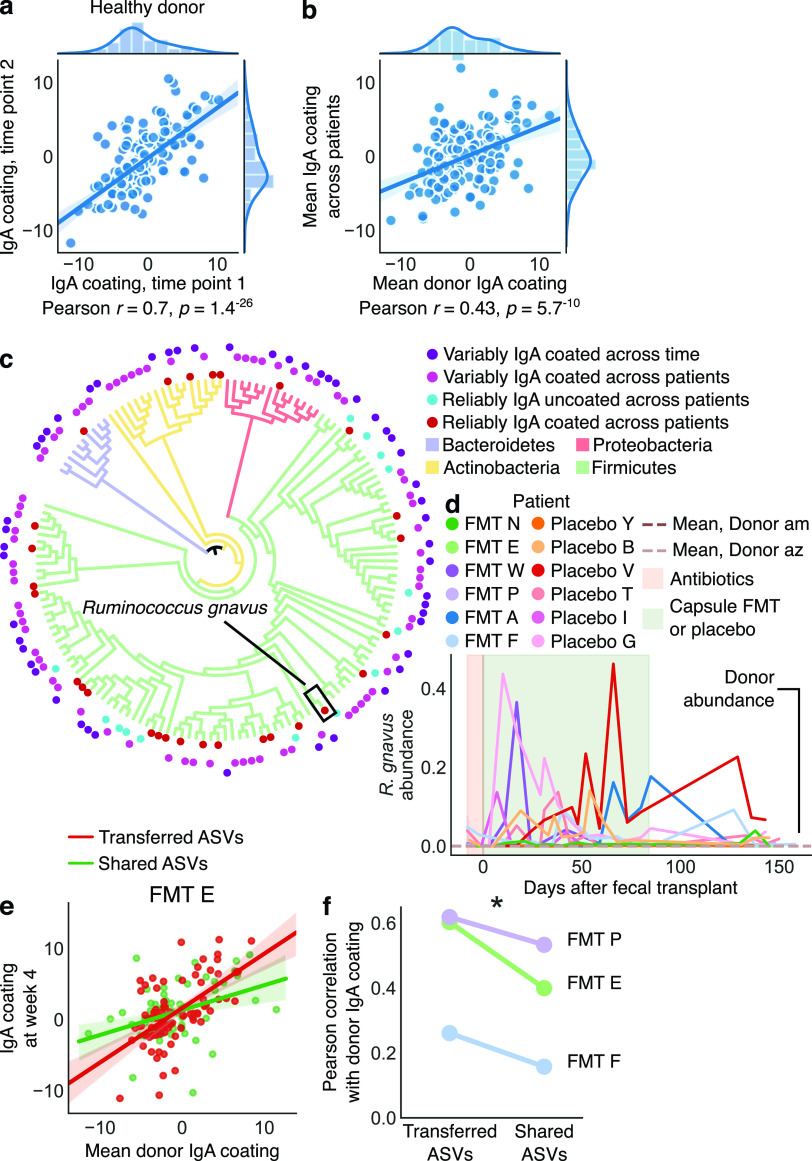
IgA coating of the gut microbiome revealed broad patterns of microbiome IgA coating and host-specific IgA responses. IgA enrichment of different bacteria was highly correlated across different samples from a healthy donor (a) and even well correlated between the same donor and the patients’ baseline samples (b). In panels a and b, each point is a bacterial ASV, and the *x* and *y* axes represent the IgA coating score of that ASV in different samples. (c) Phylogenetic tree containing bacteria that were identified as reliably IgA coated across patients (inner red circles), IgA uncoated across patients (blue circles), or variably IgA coated or uncoated either across patients or within a patient across time (outer purple circles). Tree branches are colored by phylum, and branch lengths do not reflect evolutionary distance. (d) Relative abundance of Ruminococcus gnavus in our patient cohort. (e) Correlations of IgA enrichment in shared and transferred bacteria indicated stronger correlation in transferred bacteria, which are more likely to be exact matching strains. (f) This pattern was observed in all patients receiving transplants from Donor am. See also Fig. S9.

If IgA coating of different bacteria were the same across all patients and donors, then the likelihood of successful transfer of IgA coating would be very high. We therefore first sought to determine whether overall IgA coating of bacterial ASVs differed among patients. We found that IgA coating scores of bacteria in two samples taken at different time points from one of our healthy donors were highly correlated (Pearson *r *=* *0.7, *P < *1e^−25^) (Fig. 5a). To further explore the consistency of this result, we performed IgA-seq using a magnetic bead protocol (see Materials and Methods) on stool samples from three time points from six different healthy donors (see Fig. S9a and b). We found that in the majority of cases, these healthy subjects had similar IgA coating at different times (Pearson *r *>* *0.7), although for a couple of samples this pattern did not hold, potentially suggesting that IgA coating of bacteria in apparently healthy subjects may vary occasionally over time. We then compared the mean IgA coating in samples from Donor am with the mean IgA coating scores across all patients’ baseline samples and found moderate correlation (Pearson *r *=* *0.43, *P < *1e^−9^, Fig. 5b). The strength of this correlation (donor samples versus baseline samples) for individual patients, however, varied considerably (Pearson *r *=* *0.1 to 0.7, Fig. S9c and d), indicating that bacterial IgA coating can vary greatly across patients. This finding raised the prospect that transferred bacteria may not retain immune interactions across hosts. Some studies have suggested that IgA might target blooming or abundant bacteria to promote homeostasis (41, 42), but we did not observe a correlation between a bacterium’s IgA coating and its abundance, variance, or bimodality (see Fig. S9e to h).

10.1128/mBio.00975-21.9 FIG S9 Related to Fig. 5: IgA coating of gut microbes. IgA coating of bacteria in healthy donors is often stable across multiple time points. Using a protocol based on magnetic bead selection of IgA (1), we analyzed stool samples from three time points from six different healthy donors from the OpenBiome stool bank. We found that the IgA coating of gut bacteria was remarkably stable over time in these healthy donors (time between samples varied from 6 to 73 days). (a) Example Pearson correlations between time points from each of six donors. (b) Pearson’s *r* values for all time-point comparisons within each donor. Some time points did demonstrate variation, with correlation coefficients under 0.6. (c to h) Some patients’ baseline samples had much greater correlation of IgA coating with a healthy donor than others’ samples. (a and b) Line plots (a) and corresponding Pearson *r* correlation values (b) for each patient at baseline. (c to e) IgA coating scores (a.k.a. IgA enrichment scores) did not correlate with a bacterium’s abundance (c), variance (d), or bimodality (e). (f) IgA coating scores from each sample largely followed a normal distribution. Download FIG S9, TIF file, 1.9 MB. Copyright © 2021 Chu et al. 2021 Chu et al. https://creativecommons.org/licenses/by/4.0/ This content is distributed under the terms of the Creative Commons Attribution 4.0 International license.

We then sought to identify bacteria that were strongly IgA coated or uncoated across all patients and samples, because perhaps these bacteria would display similar immune interactions when transferred across hosts. Indeed, we found 28 bacterial ASVs (*P < *0.0001 by a permutation test) that were reliably and strongly IgA coated across patients and within patients through time, including before and after FMT (see Materials and Methods). These bacteria were a phylogenetically diverse group comprising organisms from all major phyla (Fig. 5c; see also Table S7), including known commensals (e.g., *Bacteroides*) and many taxa that transferred from donors to fecal transplant recipients (e.g., *Bacteroides* and *Lachnospiraceae*; see Table S7). We also identified some Proteobacteria (including known opportunistic pathogens) and Ruminococcus gnavus—as well as the closely related *R. torques—*as reliably IgA coated in patients and donors (Fig. 5c; see also Fig. S10 and Table S7). Although we did not observe *R. gnavus* transfer from donors to patients, we found significant blooms of this bacterium (up to 40% relative abundance) in our patient cohort; the species was essentially absent in our donors (Fig. 5d). This finding is in keeping with those of other research groups, who have reported that *R. gnavus* blooms specifically in patients with IBD (43). These results further suggest that *R. gnavus* might play a role in immune dysregulation in IBD, likely warranting additional study.

10.1128/mBio.00975-21.10 FIG S10 Phylogenetic tree of bacteria showing the IgA enrichment across samples and patients. The phylogenetic tree was constructed on the basis of 16S sequences. Each column is a heatmap corresponding to each patient, indicating the IgA coating score for that bacterium at a given time point (not shown here because of size). Download FIG S10, PDF file, 0.1 MB. Copyright © 2021 Chu et al. 2021 Chu et al. https://creativecommons.org/licenses/by/4.0/ This content is distributed under the terms of the Creative Commons Attribution 4.0 International license.

Bacteria that were reliably uncoated by IgA across patients (*n *=* *14, *P < *0.0001 by a permutation test) were less phylogenetically diverse and tended to represent known commensals. All of these bacteria were *Firmicutes*, including known butyrate producers and immuno-modulatory taxa like *Faecalibacterium*, *Alistipes*, *Roseburia*, *Oscillibacter*, *Butyricicoccus*, and other *Lachnospiraceae* species (Fig. 5c; see also Table S7). On the basis of these results, we hypothesize that being uncoated by IgA may be more specific than being coated. Together, these data hint that bacteria eliciting strong or very low IgA responses across individuals could play important roles in regulating host gut immunity and that in some cases transferring these bacteria can replicate microbe-host immune interactions in a new host.

### Strain specificity revealed the transfer of host immune interactions in fecal transplant.

We observed a final category of diverse bacteria that were sometimes IgA coated and sometimes uncoated across different individuals or even within the same individual across time (Fig. 5c; see also Fig. S10 and Table S7). If confirmed, such variable immune effects across patients could complicate FMT treatment, which currently assumes the transfer of microbial functions from donors to patients. If the same microbe can elicit an IgA response in one person while avoiding it in another—which may lead to proinflammatory or anti-inflammatory responses (44)—then FMT may be less effective and less predictable than assumed at transferring immune function.

We hypothesized that variable IgA responses could stem from two processes: (i) divergent host immune responses or (ii) strain specificity of IgA coating. If the second process were responsible, a given bacterium would be IgA coated or uncoated because each patient would be responding differently to different strains of bacteria with identical 16S sequences, the genetic marker for our IgA-seq data. But each of these patients would nevertheless respond in the same way to the exact same strain, and thus transfer of the same strain across patients would transfer a similar IgA response.

To establish the role of strain specificity in explaining variably IgA-coated bacteria, we examined two bacterial subsets in fecal transplant recipients: bacteria that were shared by donor and patient and bacteria that transferred from the donor to the patient after a fecal transplant (see Materials and Methods). Thus, for each bacterial 16S sequence, the first subset included a potential mix of donor and patient strains, while the second subset likely contained only one strain from the donor that then transferred to the patient. We found that the subset of strains transferred from the donor (exact strain matches) had higher correlations of IgA coating scores than bacteria shared between the donor and recipient (mixed strains) (Fig. 5e); this pattern held across three patients who received fecal transplants from a single donor (Fig. 5f).

These results suggest that exactly matching strains may trigger more-similar immune responses than do mixed strains with identical 16S sequences, indicating that variable IgA coating of bacteria could in part be explained by strain specificity. Although in one patient the correlation of IgA coating scores of exactly matching strains was still weak (Pearson *r *=* *0.3), this finding further established that immune functions of transferred microbes may be broadly replicated in fecal transplant recipients, bolstering the prospects of engineering the gut microbiome to modulate host immunity and disease.

## DISCUSSION

### Microbial strains exhibit a range of colonization dynamics.

Our results illustrated a range of microbes and functions that can persistently colonize fecal transplant recipients. Only a small subset of rare bacteria appeared to never transfer (see Table S4), suggesting that essentially all bacteria can be transferred between people. This finding reaffirms that the gut microbiome can be clinically engineered by transplanting whole gut microbial communities. It remains unknown, however, whether more-targeted therapeutics using synthetic communities will show the same ability to colonize recipient hosts.

For fecal transplants or other targeted microbial therapeutics to have a clinical effect, colonized microbes must persist at sufficient abundance. We observed a variety of fates of colonizing microbes in each patient over time—from wholesale colonization and persistence of new taxa to fleeting passage of individual strains. A significant fraction of microbes that colonized recipients for multiple weeks later disappeared, suggesting that even though many bacteria can colonize a patient temporarily, competition, nutritional requirements, or immune system interactions may hamper persistence. This problem might be addressed by administering fecal transplants along with other treatments aimed at maintaining colonized bacteria (e.g., prebiotics and maintenance therapy) (42, 45, 46).

Coexisting conspecific strains also showed a range of competitive dynamics: in some patients donor strains dominated endogenous strains, while in others, endogenous strains remained more abundant. Competitive dynamics like these may contribute to variable clinical responses to whole-gut microbiota transplantation and are likely to play an even greater role in more-targeted microbial therapeutics, whose efficacy hinges on the dynamics of a small number of strains.

In addition, patient FMT A offered an example of how patient health can affect the colonization of donor bacteria. After more than a month of stable colonization, this patient lost a large portion of transferred strains in a short period, which coincided with—or potentially preceded—a bloom in pathogenic bacteria and severe worsening of symptoms. This dramatic decline warns us that continued patient monitoring may be needed to maintain treatment efficacy, particularly with chronic diseases like IBD. Patients who lose colonized donor bacteria could be retreated, restarting the clock on donor-strain persistence and intended clinical effect. Furthermore, although it remains unknown which of these shifts came first—loss of donor bacteria, bloom of pathogens, or worsening of symptoms—the progressive unfolding of these events raises the possibility that real-time tracking of patient microbiomes may enable early intervention and prevention of IBD flares.

### Microbial and immune functions transfer across human hosts.

We found that specific beneficial functions transferred from donors to patients and could also persist. Many gut microbiome studies have focused on the benefits of butyrate production, for example, and we were able to track the transfer of butyrate production genes from donor to patient. But even after receiving new butyrate genes from a donor, fecal transplant recipients did not show higher butyrate gene diversity compared to placebo-treated patients. This observation suggests that it may be difficult to increase overall genetic capacity for butyrate production via fecal transplants. Of course, the diversity of genes related to a function does not necessarily reflect the activity of those biochemical pathways or the resulting amount of butyrate in the colon. Indeed, best-practice methods to measure clinically relevant butyrate production are not well established, since butyrate is continually produced by the microbiome and absorbed by the epithelium. Instead, it may be more fruitful to focus on which butyrate-producing organisms are present (Are some microbes more productive than others?) and which nutrients (e.g., dietary fibers) are available to those bacteria. Examining how colonization, persistence, and environmental context alter the activity of transferred gut bacteria is likely to bring us closer to understanding the pharmacokinetics of gut microbiome engineering.

We found that fecal transplants can also transfer unintended functions (e.g., antibiotic-resistance genes and virulence factors). To date, no evidence suggests that such unintended transfers have appreciable clinical effects (1, 47), but possibilities must be considered, particularly since antibiotic resistance can transfer among gut bacterial species (47, 48). Although it is probably impossible to purge an intact fecal community of all antibiotic resistance, targeted microbial therapeutics may be able to minimize or avoid it.

In the context of IBD, the function most critical to transfer and persist in the patient is the gut microbiome’s immune function. Our identification of numerous reliably and strongly IgA-coated or -uncoated bacteria across all patients and donors indicated retention of immune interactions across hosts. Strongly IgA-coated bacteria included IBD-associated bacteria (*R. gnavus* and E. coli), as well as known commensals (*Bacteroides* and *Blautia*)—a finding that complicates the frameworks of research suggesting that IgA-coated bacteria are largely pathogenic and inflammatory (17, 38). It may be that IgA coats any bacterium colonizing the gut mucosa, whether friend or foe. This speculation fits with recent reports of *Bacteroides* commensals using IgA to colonize the mucosa (39) and with the observation that IgA and gut bacteria tend to concentrate in the outer layers of mucus in mice (49).

Furthermore, we established that IgA coating of bacteria can be transferred across human hosts, leading us to speculate that transferring gut microbes may be broadly effective in triggering specific and nonspecific IgA coating and immune pathways. Because IgA can be polyreactive—a single immunoglobulin can recognize multiple antigens (50)—this transfer may not reflect a specific adaptive immune response of the recipient host but, at minimum, reflects that the transferred microbe retained the same IgA coating. In addition to bacteria that were reliably IgA-coated or -uncoated, many bacteria were variably IgA coated across patients, hinting that potential host specificity of immune interactions could complicate clinical responses to fecal transplants. We found that this variability was in part due to the strain specificity of IgA coating: strains that transferred from donor to patient tended to have similar patterns of IgA coating. This specificity seems to contrast with previous reports of polyreactive IgA activity in the mouse small intestine (50). It is highly unlikely that this signal resulted from the IgA coating of bacteria in the daily capsules, because of their small dosage (one capsule per day) and the necessity for those bacteria to pass through the small and large intestines.

It is further possible that a donor’s immune context may play a role in the transfer of IgA coating from donor to patient, either because of donor-specific immune responses or other donor-specific factors like diet or microbial community. For example, a particular bacterial strain might express different surface receptors depending on the nutrients in a host’s diet, which may then alter what would otherwise be identical immune interactions (44). We further speculate that an IgA-coated bacterium from a donor, transferred into a patient, may retain its IgA coating, not because the patient innately coats that bacterium but because the patient’s immune system “learns” the coating pattern from the donor’s IgA. In such a scenario, IgA coating and immune function may display an “inertia” when transferred between hosts. Additional study into the immune factors that generate transferable and variable IgA responses would illuminate our ability to manipulate host immunity via the gut microbiome.

In summary, our study offers a first look at the dynamics of colonization and persistence of microbes, their metabolic functions, and their immune functions in UC patients treated with FMT. Our dense time series analysis revealed surprising complexity in microbial transfer and emphasized that for chronic diseases like IBD, continuing patient care may be necessary to maintain newly colonized bacteria. Our observations of broad transfer of microbes and their functions further demonstrate the power of FMT to alter a patient’s gut microbiome and begin to set the stage for developing targeted drugs that introduce and maintain specific microbes and functions to treat disease.

## MATERIALS AND METHODS

### Clinical cohort and sample collection.

We obtained samples from a clinical cohort recruited at the University of Vermont Medical center in Burlington, VT (24). Patients collected approximately weekly stool samples at home or in the clinic, storing samples in RNAlater solution (Thermo Fisher) and mailing them to a processing facility at OpenBiome in Somerville, MA. During clinical evaluations at University of Vermont Medical Center, fresh stool samples were also collected at baseline and at 4, 12, and 18 weeks after the initiation of FMT. Fecal samples were either produced during the clinical visit or less than 24 h before the appointment. The latter samples were kept in a fridge and then transported on wet ice to the clinic. At the clinic, fresh samples were then mixed with a glycerol buffer (1× PBS [phosphate-buffered saline], 25% glycerol, 0.05% l-cysteine) and stored at −80°C (24).

### DNA extraction and sequencing.

We triple-washed RNAlater from samples in 1× PBS and extracted DNA using a MoBio Powersoil DNA extraction kit. 16S rDNA libraries were prepared and sequenced by the Broad Institute Genomic Platform, using the Earth Microbiome Project protocols and paired-end 250-bp reads on an Illumina MiSeq (51). Shotgun metagenomic libraries were likewise prepared by the Broad Institute using Nextera protocols and sequenced on an Illumina NextSeq.

### IgA sequencing.

Samples were processed as described previously (17). We centrifuged glycerol-stored stool samples at 50 × *g* at 4°C for 15 min and then washed them three times in 1 ml PBS/1% BSA at 8,000 × *g* for 5 min. We collected the presort fraction as 20 μl after resuspension before the final wash and stored the washed samples at −80°C. We then resuspended the cell pellet in 25 μl of 20% normal rat serum (Jackson ImmunoResearch) in PBS/1% bovine serum albumin (BSA) and incubated the samples for 20 min on ice. After incubation, we added 25 μl of 1:12.5 α-mouse-IgA-PE (eBioscience; clone mA-6E1) to each sample and incubated samples on ice for 30 min. Finally, we washed samples three times in 1 ml PBS/1% BSA, resuspended them in PBS/1% BSA, and transferred them to blue filter-cap tubes (VWR 21008-948) for flow sorting. We sorted an average of 50,000 cells from the IgA-positive and IgA-negative bacteria into sterile microcentrifuge tubes on the BD FACSAria II at the MIT Koch Institute Flow Cytometry Core (Cambridge, MA). We then centrifuged the samples, removed the supernatants, and resuspended the pellets in a final volume of 10 μl of sheath fluid. Samples were stored at −80°C until DNA library prep, in which 2 μl (∼10,000 cells) was used directly as the template for PCR.

### Data analysis.

We analyzed 16S data using Qiime2 (26), DADA2 (27), and custom Python scripts. We assigned taxonomic labels to 16S sequences using the SILVA database (52). We quantified the abundance of microbial species from shotgun metagenomic sequencing using MetaPhlAn2 (28). To visualize changes in alpha and beta diversity, we calculated the mean values of samples within 5-day windows and compared these values across treatments using a Mann-Whitney U test.

To track the sources of various bacteria, we defined all bacteria observed in any of a patient’s baseline samples and in the donor sample as “Shared,” all other bacteria present in baseline samples as “Patient,” all bacteria absent from baseline but shared with the donor as “Donor,” and finally all others as “Unknown.” We defined “persistent” colonization as a bacterium (ASV or metagenomic species) that transferred exclusively from a donor and appeared in at least three samples after the initiation of FMT and remained present in at least one follow-up sample at ∼18 weeks after initial transplant. We defined “temporary” colonization similarly, except that such bacteria did not appear in any follow-up samples.

Assigning sources using this strategy has its limitations. Because many strains within common bacterial taxa have identical 16S sequences, 16S-based techniques may register many unique strains as a single ASV. For example, in the case of E. coli, all transplant and placebo-treated patients and the donors shared a single E. coli ASV, but it is highly unlikely that every patient in fact shared the same E. coli strain. Thus, our strategy may occasionally falsely identify the source of a given ASV. Consequently, we focused on the overall frequency (occurrences) of ASVs from different sources, instead of the abundance of each ASV. This approach minimizes the signal from highly abundant but potentially incorrectly identified ASVs, since it weights all ASVs equally.

To quantify the transfer of bacterial functions, we used ShortBRED (53) to determine the abundances of genetic functions of interest, including butyrate biosynthesis (54), mucin degradation (55), glycoside hydrolase activity (56), antimicrobial resistance (57), and virulence factors (58). We quantified the abundance of quinolone resistance in baseline samples and in the 10 days immediately after antibiotics stopped, and we compared these abundances using a Mann-Whitney U test. We visualized the abundance of genetic functions in FMT and placebo patients using the same five-day windows as described above. We identified the sources of antibiotic resistance genes and virulence factors in the same way we identified bacterial sources.

To quantify the transfer of strains, we used two strategies: one based on flexible genome content and the other on single-nucleotide variants. For the first, we used a strategy similar to that described previously (34). Briefly, we mapped metagenomic reads from each sample to reference genomes for each species using BWA (59) and quantified the number of reads mapping to each unique 1,000-bp segment of the reference sequence. To compare the strains in two samples, we then compared the read depths in each sample across all 1,000-bp segments. We identified a strain match as those comparisons for which no segment with a read depth greater than the median for that sample was entirely absent from the other sample (Fig. S5). Comparisons that did not meet these criteria were called ambiguous. Comparisons where either sample had a median read depth less than 5 were not considered because of insufficient abundance and read depth. To reconstruct the individual contributions of strain haplotypes, we used StrainFinder (31). To build the input alignments for StrainFinder, we used BWA (59) to align metagenomic reads from each sample to a database of AMPHORA genes (60)—a set of single-copy, universally carried bacterial genes—from various gut bacteria. We used SAMtools (61) to tally the nucleotide identities found at each position and filtered stringently to remove reads with poor mapping quality, rare alleles, and sites with inordinate read depth. To provide greater depth of reads to StrainFinder’s maximum-likelihood model, we combined reads from samples across our time series as described above. We considered only genomes with a median read depth exceeding 50 in at least two samples. StrainFinder outputs the relative contributions of different strains to the abundance of a given species, so we normalized these values using the median read depth in each sample to better reflect the relative abundances of each strain across samples.

To understand host-immune interactions, we analyzed 16S data from IgA-seq using Deblur (62). We calculated IgA coating scores as the log_2_-fold change between the IgA-coated and -uncoated fractions. We observed that IgA coating scores somewhat followed a normal distribution (see Fig. S6f); thus, for each sample, we categorized strongly IgA-coated or -uncoated bacteria as those bacteria that were more or less than the mean ± 1 standard deviation. To identify bacteria that were reliably IgA coated or uncoated across all samples or across all patients, we used a one sample *t* test, with an FDR-adjusted *P* value of <0.1. We further evaluated whether these reliably IgA-coated or -uncoated ASVs were statistically significant by a permutation test, in which we randomly shuffled the ASV labels in each sample 10,000 times and counted the occurrences of the same or higher number of reliably IgA-coated or -uncoated ASVs. We constructed a phylogenetic tree of 16S sequences using FastTree (63) and visualized it using iTOL (64).

### Data availability.

We deposited raw sequence files of 16S, metagenomic, and IgA-sequencing results in NCBI’s SRA database (BioProject PRJNA475599). Metadata are included as supplementary tables which can be found at the accompanying GitHub repository (https://github.com/nathanieldchu/uc_fmt/tree/master/supplemental_materials). Additional 16S ASV tables and metagenomic species tables, as well as the code used to generate the figures and analyses, can be found at https://github.com/nathanieldchu/uc_fmt.
