# Supplementary figures and images for: Dynamic Colonization of Microbes and Their Functions after Fecal Microbiota Transplantation for Inflammatory Bowel Disease
Source: mBio. 2021 Jul 20;12(4):e00975-21. doi: 10.1128/mBio.00975-21 (PMC8406238; doi:10.1128/mBio.00975-21)

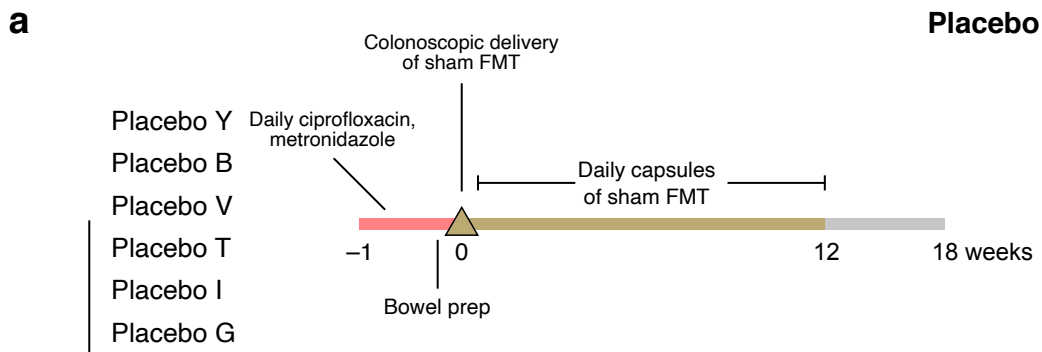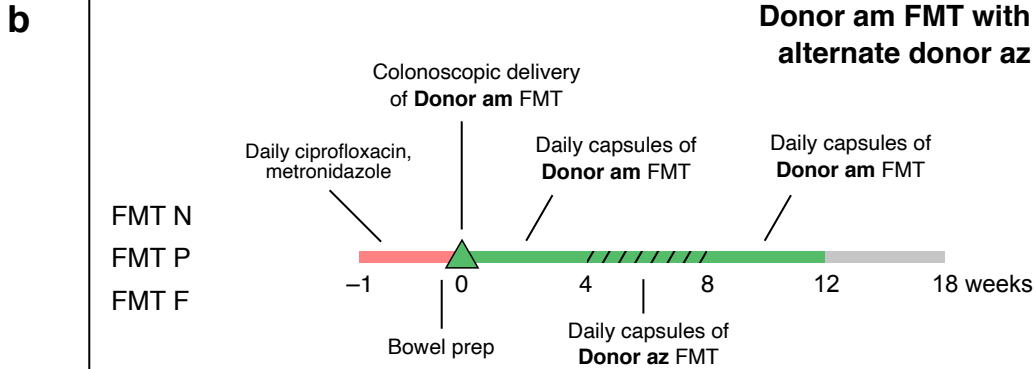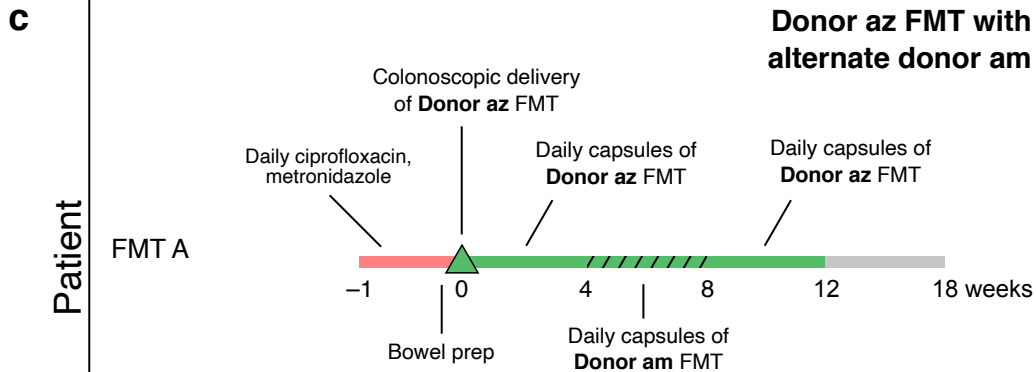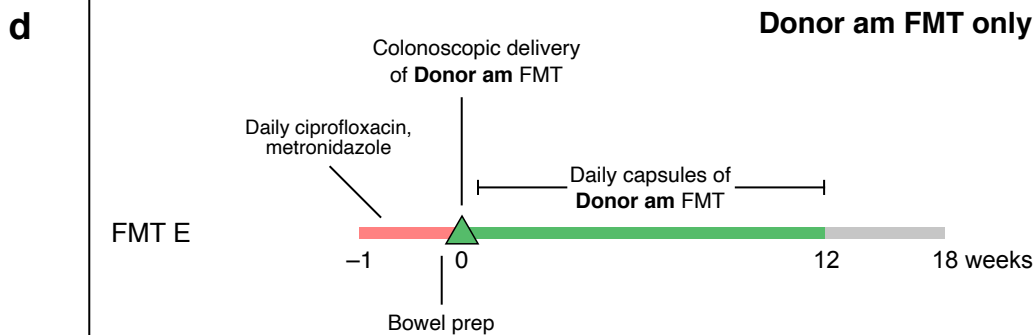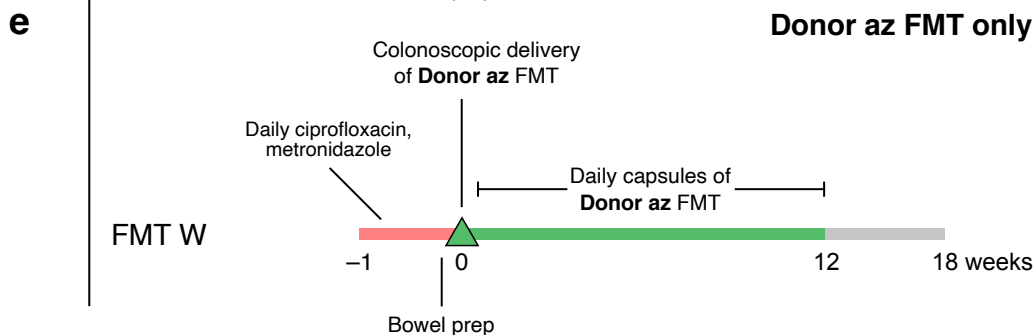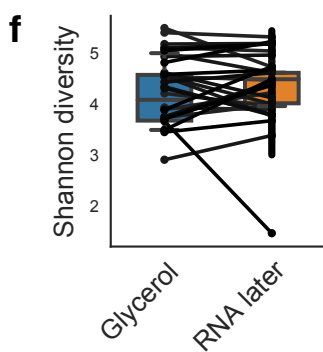

Supplement: FIG S1 [file mbio.00975-21-sf001.pdf]

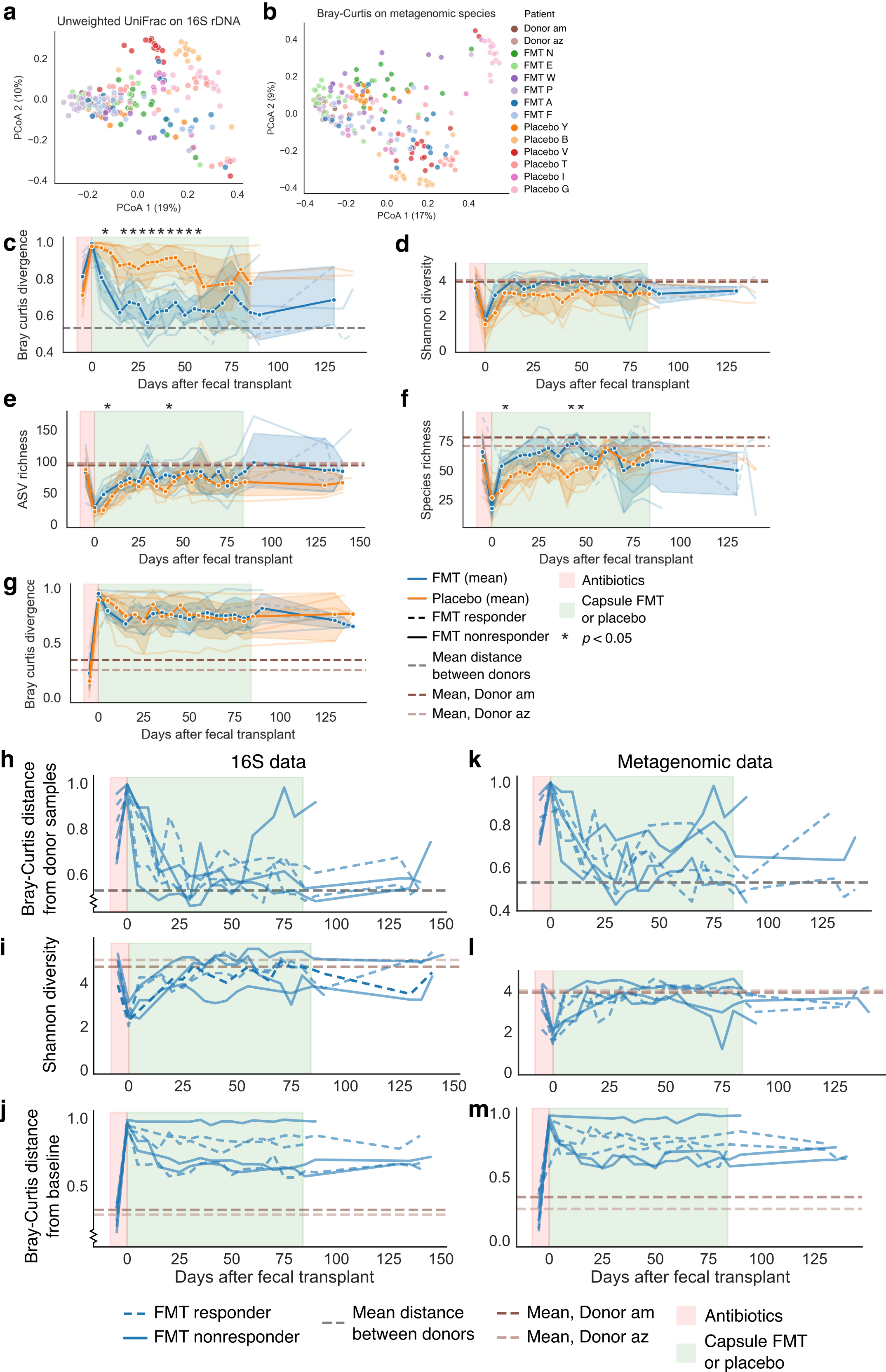

Supplement: FIG S2 [file mbio.00975-21-sf002.tif]

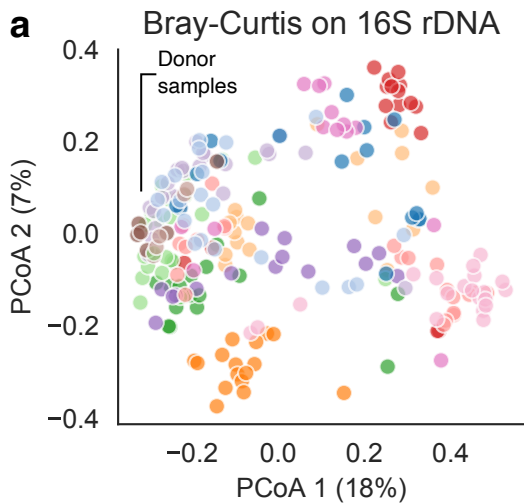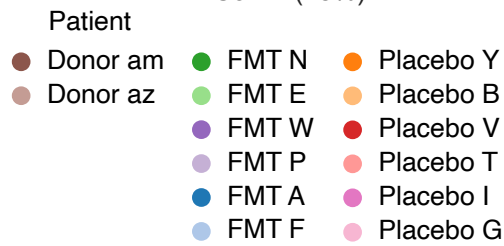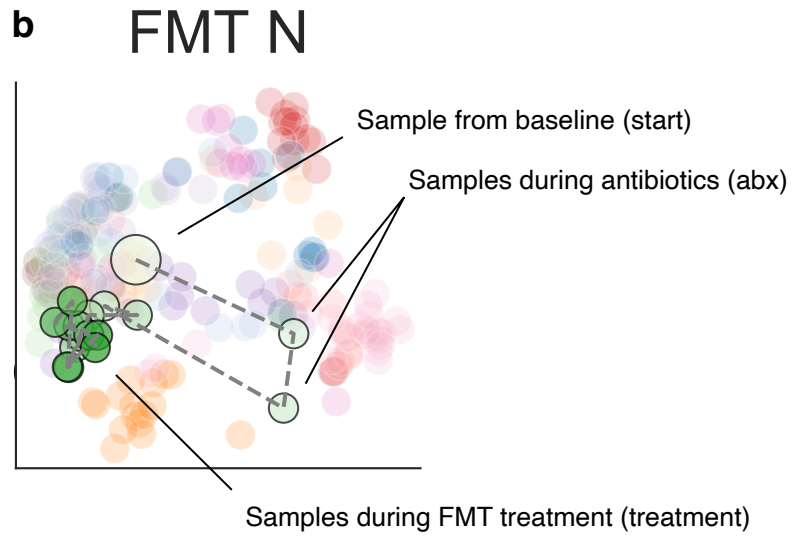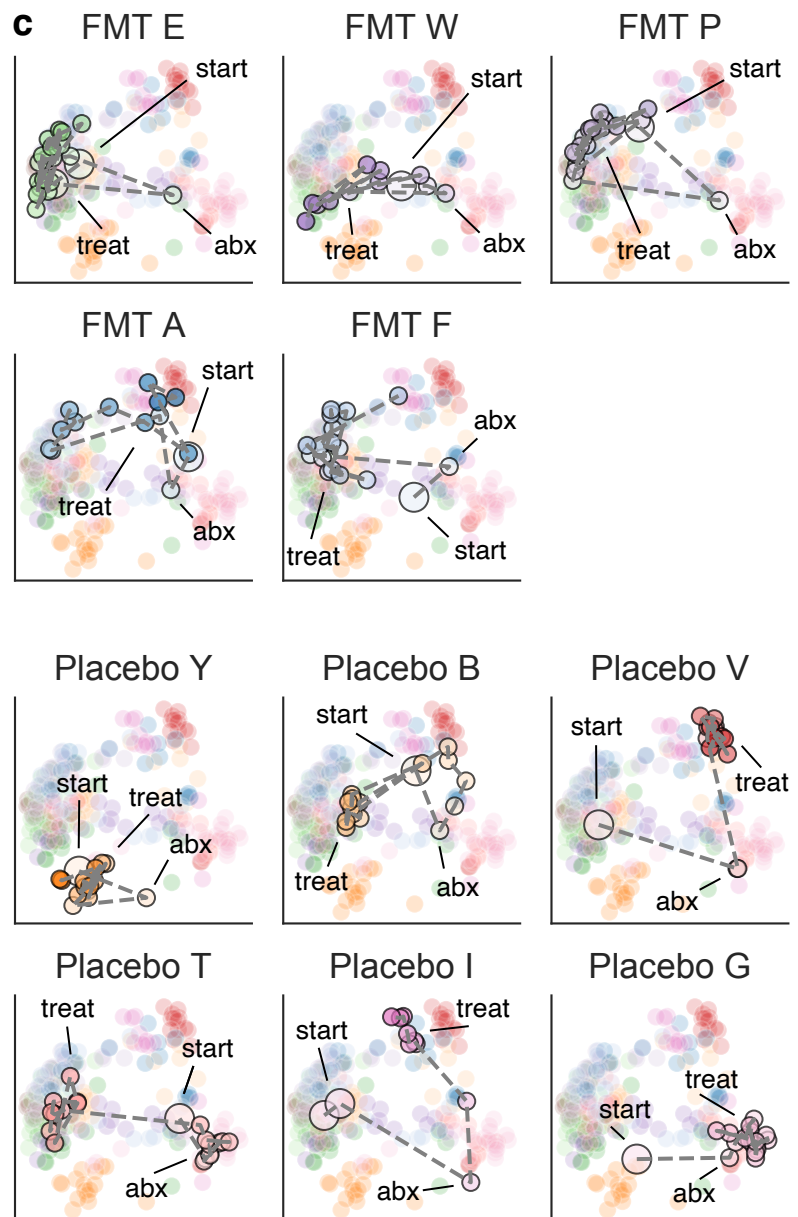

Supplement: FIG S3 [file mbio.00975-21-sf003.pdf]

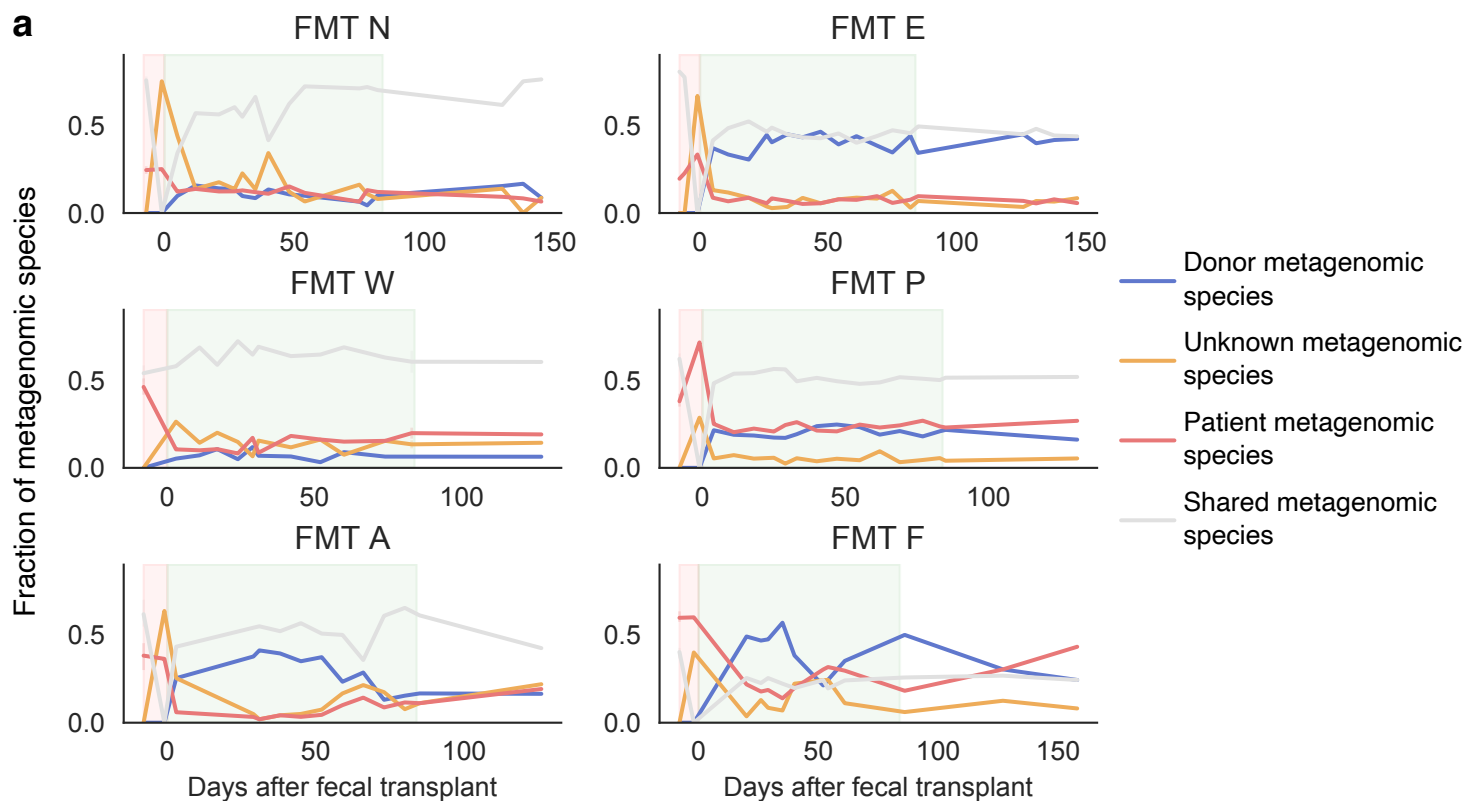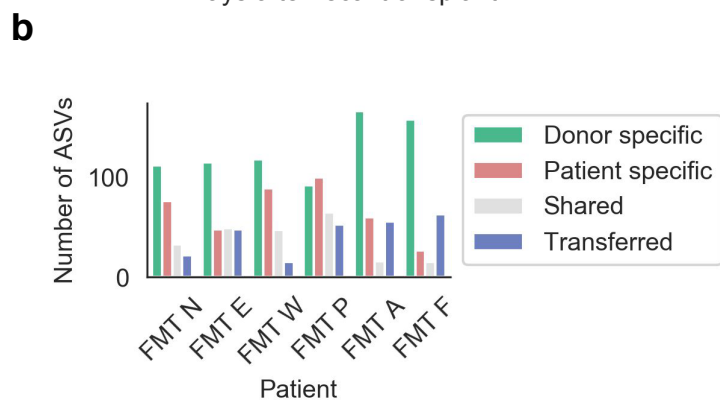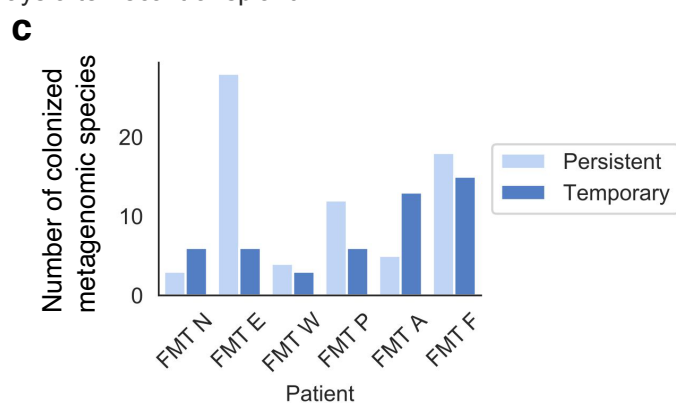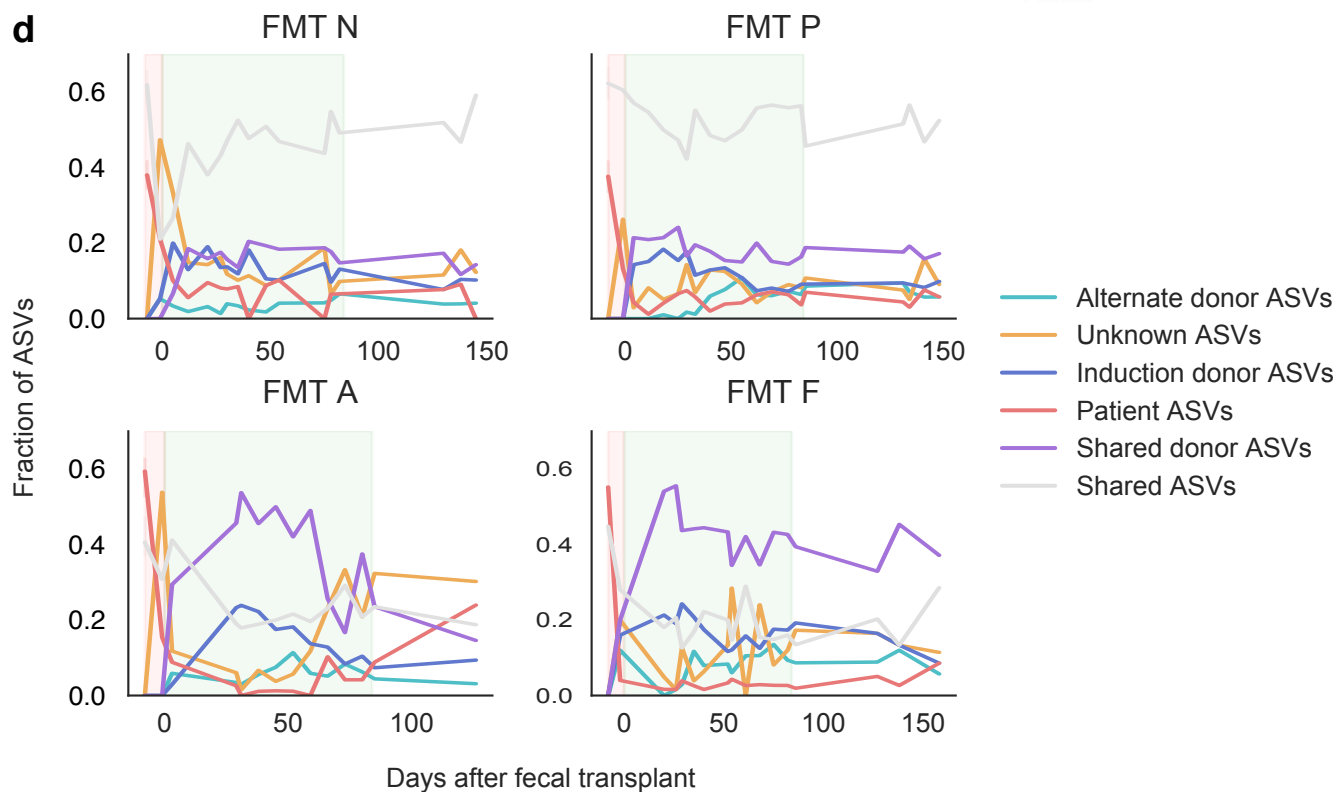

Supplement: FIG S4 [file mbio.00975-21-sf004.pdf]

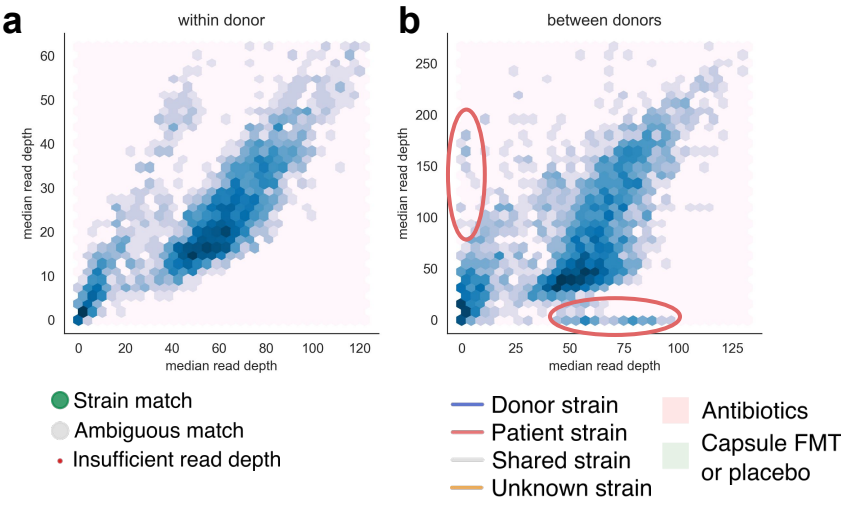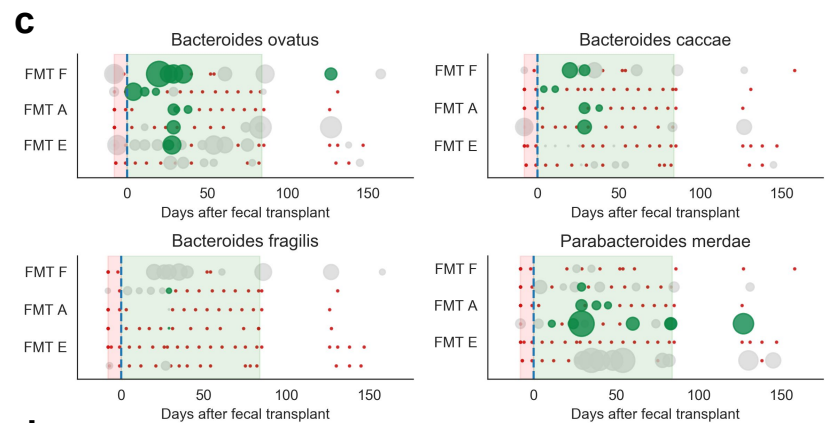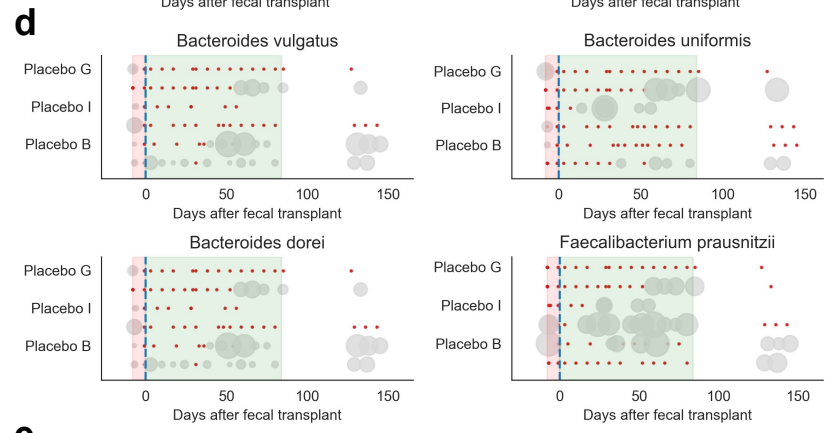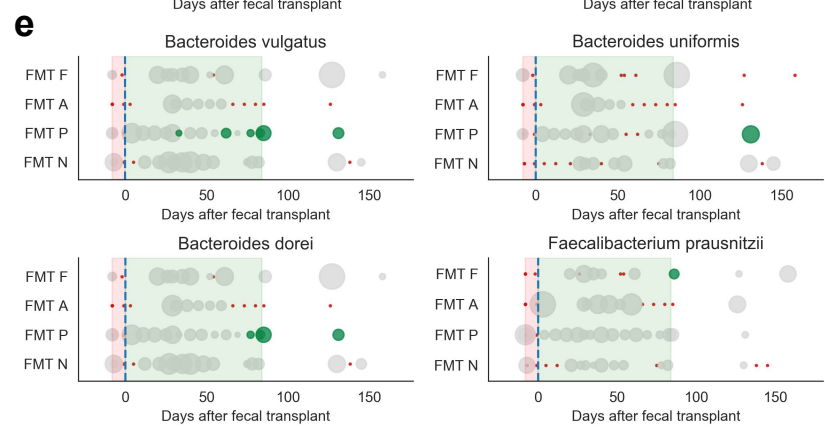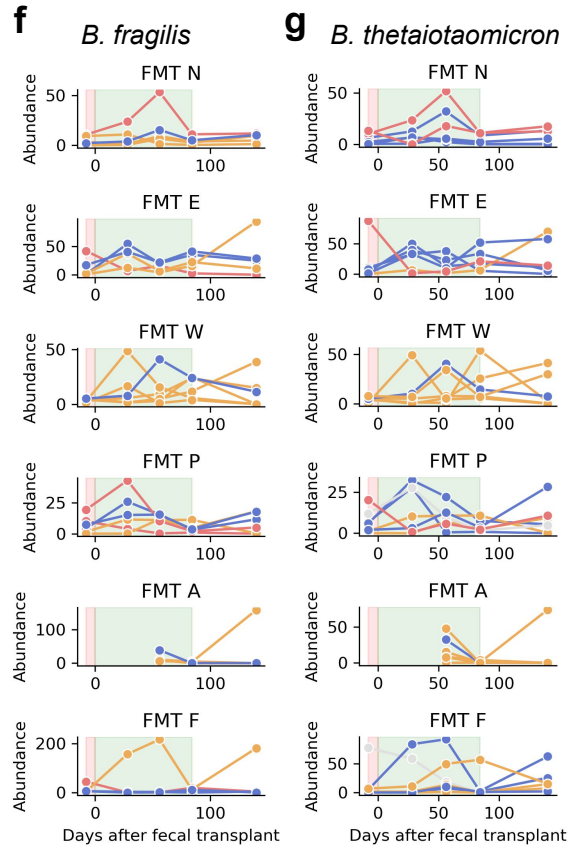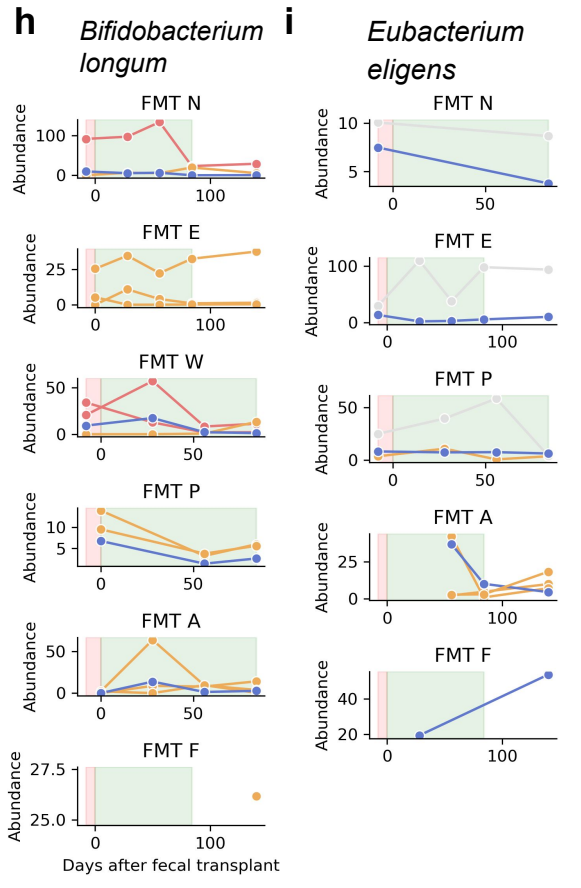

Supplement: FIG S5 [file mbio.00975-21-sf005.pdf]

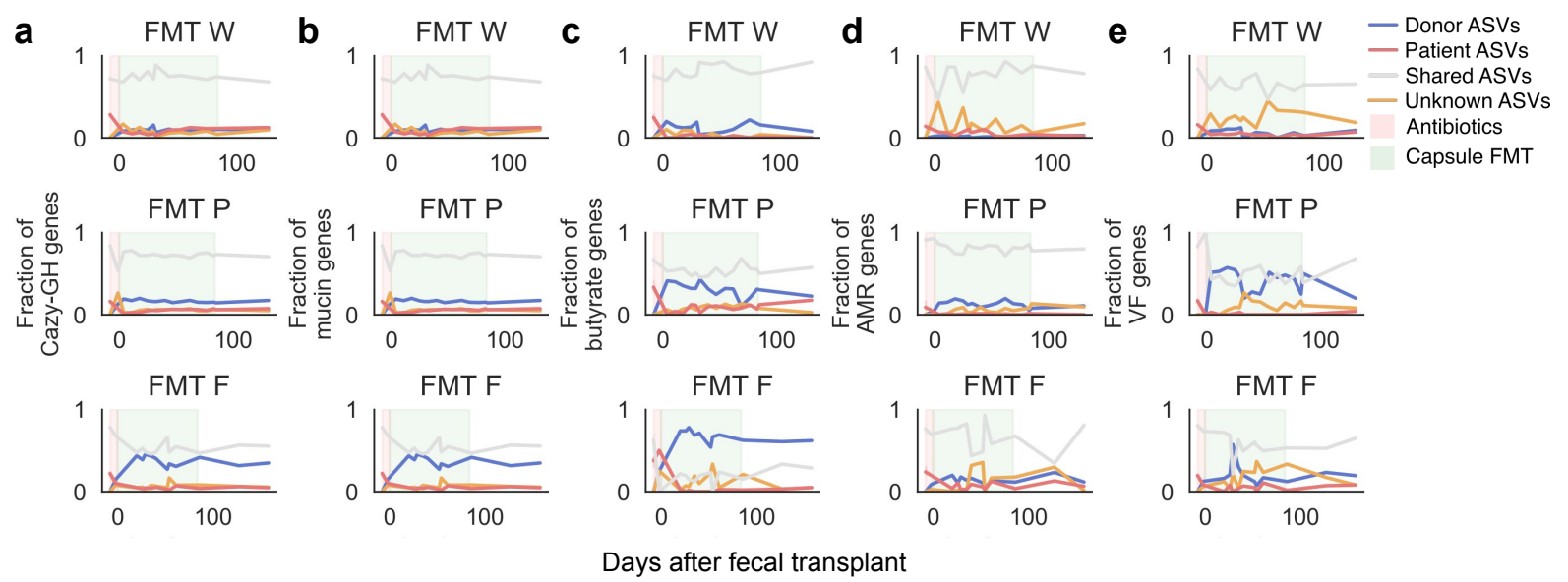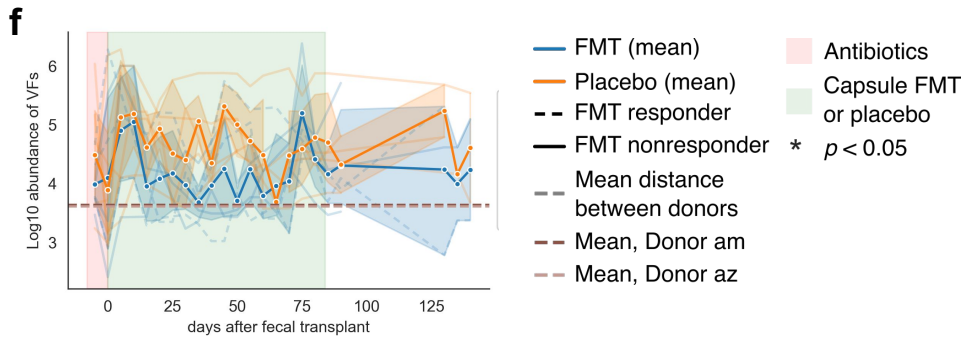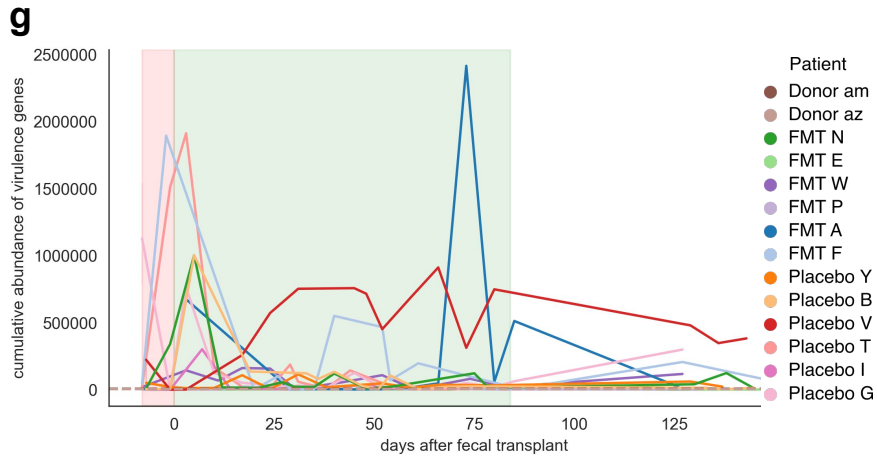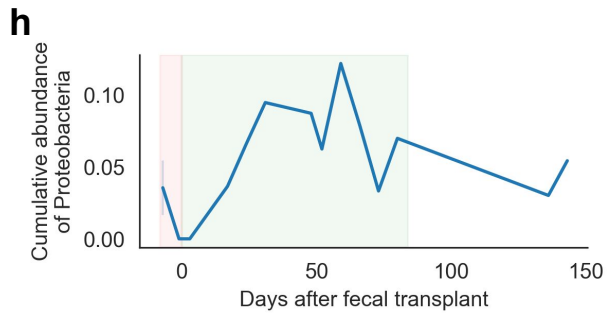

Supplement: FIG S6 [file mbio.00975-21-sf006.pdf]

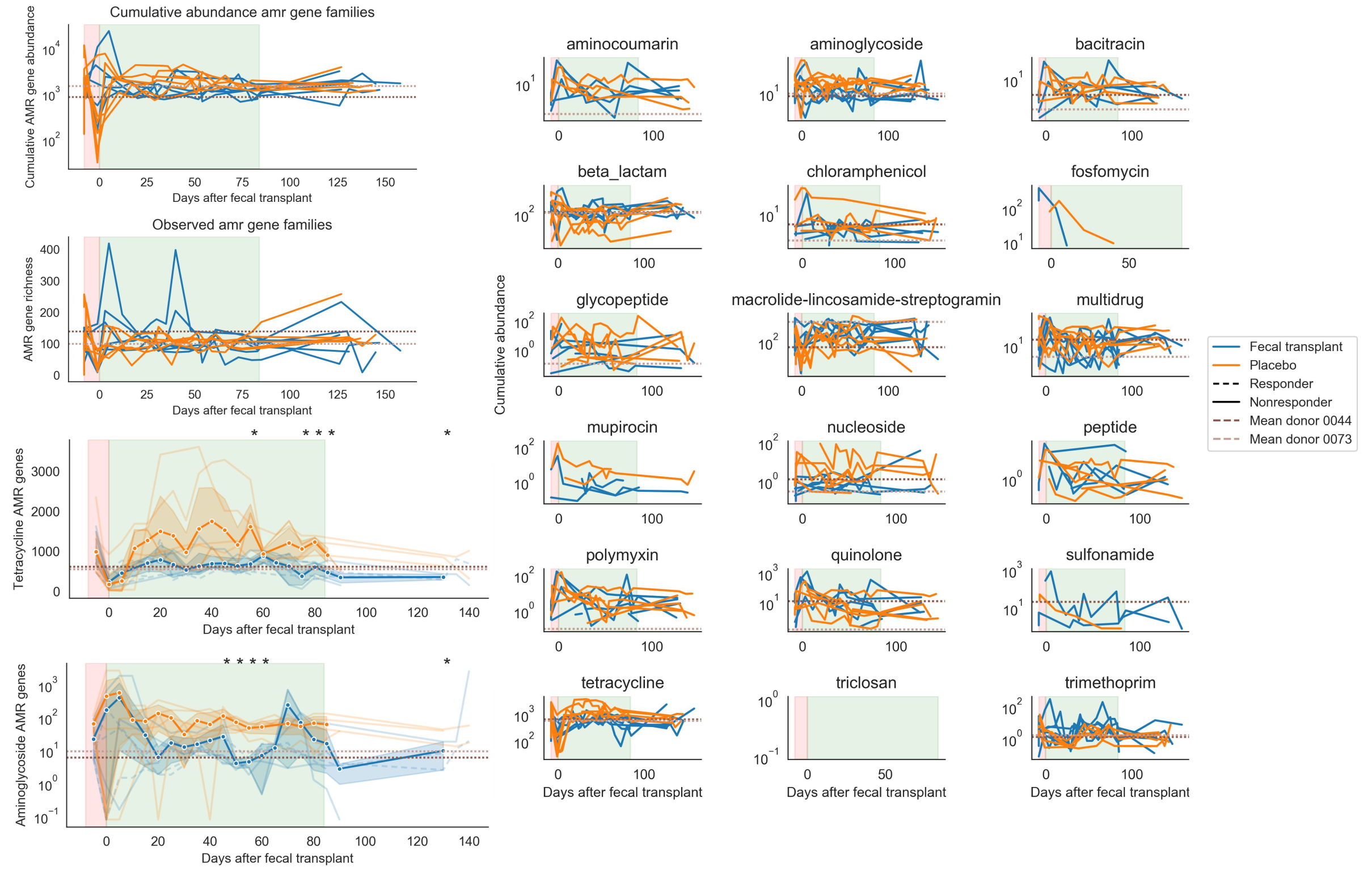

Supplement: FIG S7 [file mbio.00975-21-sf007.tif]

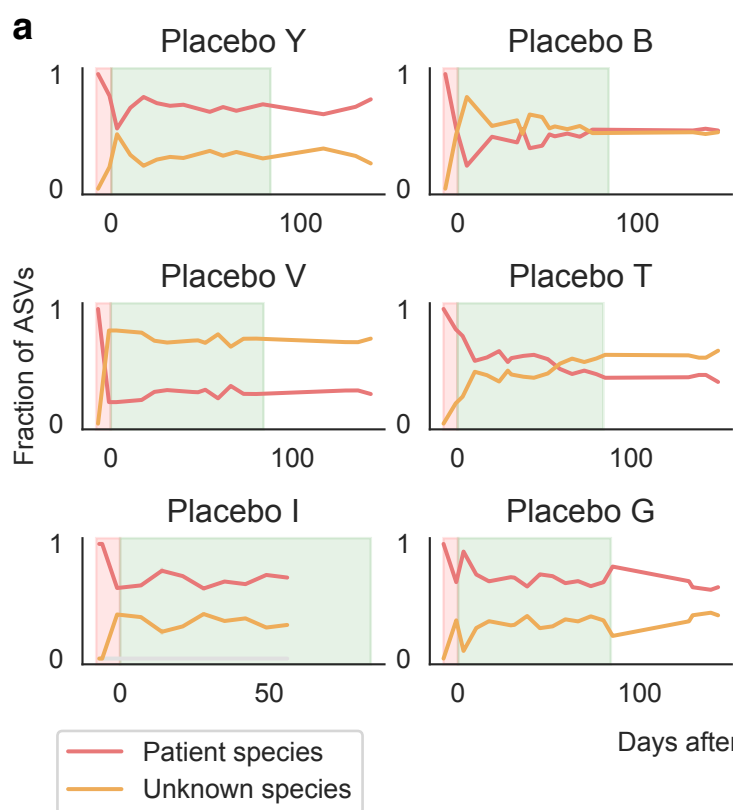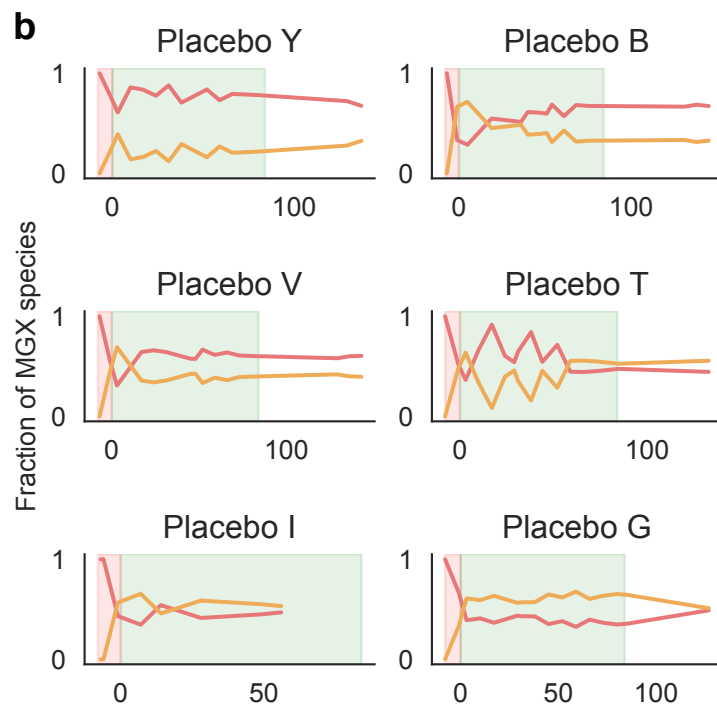

Supplement: FIG S8 [file mbio.00975-21-sf008.pdf]

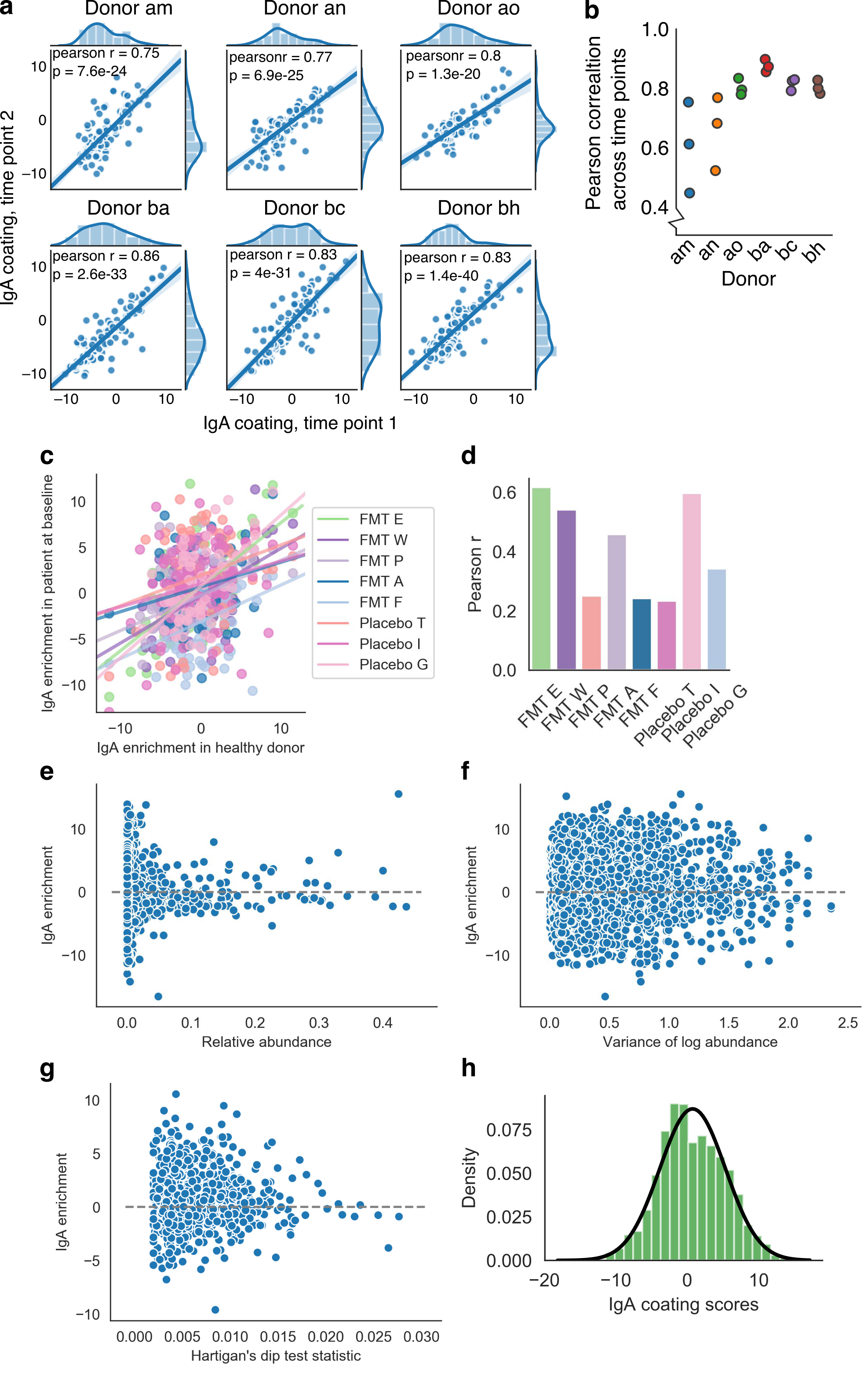

Supplement: FIG S9 [file mbio.00975-21-sf009.tif]
